# Microbial-tubeworm associations in a 440 million year old hydrothermal vent community

**DOI:** 10.1098/rspb.2018.2004

**Published:** 2018-11-14

**Authors:** Magdalena N. Georgieva, Crispin T. S. Little, Russell J. Bailey, Alexander D. Ball, Adrian G. Glover

**Affiliations:** 1 Life Sciences Department, Natural History Museum, London, UK; 2 Imaging and Analysis Centre, Natural History Museum, London, UK; 3 School of Earth and Environment, University of Leeds, Leeds, UK; 4 The NanoVision Centre, Queen Mary University of London, London, UK

**Keywords:** symbiosis, early life, chemosynthesis, fossil, pyrite, microfossil

## Abstract

Microorganisms are the chief primary producers within present-day deep-sea hydrothermal vent ecosystems, and play a fundamental role in shaping the ecology of these environments. However, very little is known about the microbes that occurred within, and structured, ancient vent communities. The evolutionary history, diversity and the nature of interactions between ancient vent microorganisms and hydrothermal vent animals are largely undetermined. The oldest known hydrothermal vent community that includes metazoans is preserved within the Ordovician to early Silurian Yaman Kasy massive sulfide deposit, Ural Mountains, Russia. This deposit contains two types of tube fossil attributed to annelid worms. A re-examination of these fossils using a range of microscopy, chemical analysis and nano-tomography techniques reveals the preservation of filamentous microorganisms intimately associated with the tubes. The microfossils bear a strong resemblance to modern hydrothermal vent microbial filaments, including those preserved within the mineralized tubes of the extant vent polychaete genus *Alvinella*. The Yaman Kasy fossil filaments represent the oldest animal–microbial associations preserved within an ancient hydrothermal vent environment. They allude to a diverse microbial community, and also demonstrate that remarkable fine-scale microbial preservation can also be observed in ancient vent deposits, suggesting the possible existence of similar exceptionally preserved microfossils in even older vent environments.

## 1. Introduction

Bacteria and archaea are an intrinsic component of modern hydrothermal vent communities, being the chief primary producers within these ecosystems and sustaining remarkable biomass in the otherwise largely resource-limited deep sea [1]. These microorganisms occupy a variety of niches at vents, including biological and mineral surfaces, hydrothermal plumes and extend into the sub-seafloor. They also constitute an important food source for grazing animals, and some bacteria form unique and important associations with vent metazoans such as large tubeworms, including ectosymbiosis, endosymbiosis and commensalism. In addition, vent microorganisms are highly diverse taxonomically, comprising many novel lineages of archaea and bacteria, especially *ɛ*-Proteobacteria [2–4].

Hydrothermal vents have been suggested as the probable environments within which life may have originated [5–7], and microbial fossils, in addition to stromatolites considered to be fossil microbial mats, constitute the earliest morphological evidence for life on Earth [8–10]. Many of the oldest proposed microbial fossils occur within hydrothermally influenced settings [11–13], yet microbial fossils from ancient high-temperature vents are extremely rare [14,15]. Filamentous structures, reported from the 3.24 Ga Sulfur Springs volcanic-hosted massive sulfide deposit (VHMS) [14] have been deemed the oldest example of thermophilic chemotrophic vent biota; however, the biogenicity of these filaments has subsequently been questioned [16].

The earliest metazoan fossils from hydrothermal vents occur in the late Ordovician to early Silurian Yaman Kasy VHMS deposit. Six species have been described from this deposit, two of which are worm tube fossil taxa. Possible fossil microorganisms, reported as 1 µm diameter holes, have previously been described from the walls of these tubes [17,18]. Nothing is known of the way metazoans inhabiting ancient vent systems would have interacted with microorganisms, but key to the interpretation of these possible fossil microorganisms are emerging data from studies of fossilization processes occurring at modern hydrothermal vent environments. These have shown that filamentous microorganisms can be preserved in micrometre-scale detail in pyrite, and can retain very fine cellular morphological structures such as septae within filaments [19]. These microorganisms are fossilized within, and adjacent to, sulfide-replaced dwelling tubes of the vent annelid *Alvinella* sp*.*, thereby demonstrating that microbial–animal interactions can potentially be observed in the fossil record, given similar preservation processes.

Here, we document in detail for the first time to our knowledge, filamentous microorganisms associated with macrofossils from the Yaman Kasy VHMS deposit. This study reveals the preservational and morphological characteristics of an essential component of the oldest known hydrothermal vent community. It also gives insights into the nature of microbial associations with ancient vent animals, and the potential for similar preservation of microorganisms within even older vent deposits.

### (a) Geological context and details

The Yaman Kasy VHMS deposit is located in the southern Ural Mountains, Russia (51.4068° N, 57.6930° E; electronic supplementary material, figure S1). The age of this deposit is not particularly well constrained owing to a lack of biostratigraphically useful fossils at the locality, but is considered be late Ordovician to early Silurian [18,20], approximately 440 Ma. The deposit comprises a lens of Cu-Zn-rich massive sulfides up to 37 m thick and 90–100 m in diameter within calc-alkaline volcanic rocks [18,20], interpreted to have formed within a back-arc basin [21]. Based on fluid inclusion analyses, the temperatures of the fluids from which sulfide minerals precipitated ranged from 103 to 371°C and did not boil, suggesting that the deposit was formed at not less than approximately 1600 m water depth [22]. Sulfur isotopic (∂^34^S) analyses of fossil, chimney and mound sulfides indicate an igneous source for positive values, and a probable bacteriogenic source for the lightest ∂^34^S values [22]. ∂^13^C carbon isotope analyses of organic material preserved in the Yaman Kasy deposit also yield values indicative of microbial fractionation, as well as biomarkers of potential microbial origin [23].

The Yaman Kasy metazoan fossils were found within the upper clastic sulfide sections of this deposit, and co-occur with colloform pyrite and black smoker (i.e. high temperature) vent chimney fragments [22]. The fossil assemblage comprises a monoplacophoran mollusc (*Thermoconus shadlunae*), a lingulate brachiopod (*Pyrodiscus lorrainae*), an ambonychiid bivalve (*Mytilarca* sp.), an indeterminate vetiagastropod, as well as two morphotypes of tubes [20]. The smaller tubes (*Eoalvinellodes annulatus*) are 0.2–3 mm in diameter, while the larger tubes (*Yamankasia rifeia*) range from 3 to 39 mm in diameter. The original tube walls are not preserved, but were probably organic in composition because many of the fossil tubes have folds or wrinkles [24]. The tube walls are now formed either of framboidal or colloform pyrite, and also retain fine details of the external tube wall ornamentation. *Eoalvinellodes annulatus* tubes often have thick walls comprising colloform pyrite that is many layers thick, and have external ornament of transverse, bifurcating wrinkles [18,20,25]. Small holes in *E. annulatus* tube walls have been interpreted as moulds of microorganisms [18]. *Yamankasia rifeia* tubes are either preserved as several (2–3) layers of pyrite, or by a single layer of colloform pyrite that is interpreted as having grown onto the outside of the tube [20]. These tubes show an external ornament of fine parallel longitudinal striations [18,20]. *Yamankasia rifeia* tubes also possess small holes in the colloform pyrite tube coatings, which have also been suggested to be microbial fossils [17,18].

## 2. Methods

Fragments of fossil *Y. rifeia* and *E. annulatus* tubes from Yaman Kasy, a subset of which are housed in the collections of the Natural History Museum, UK (NHMUK) were embedded in resin blocks and then polished (finishing with 1 µm diamond). They were subsequently examined with optical microscopy (in reflected light), then given approximately 10 nm thick carbon coating for backscattered electron imaging using an FEI Quanta 650 field emission gun (FEG)-ESEM at NHMUK. Elemental composition of mineral phases at various scales was determined through energy dispersive X-ray spectroscopy (EDS) using a Bruker Flat Quad 5060F detector fitted within the above scanning electron microscope (SEM). Tube-scale maps were collected at 12–20 kV. For micrometre-scale element maps, an accelerating voltage of 10 kV was used, and X-rays collected for 31–85 min with counts averaging 144 000–160 000 counts per second for each map. Interaction volumes for all detailed maps were estimated as 0.4 µm in diameter and 0.4 µm deep by the Bruker Esprit software used to analyse the data. Electron probe micro-analysis (EPMA) was also performed to assess the composition of pyrite directly around microfossils, and in nearby non-fossiliferous pyrite (see the electronic supplementary material, methods supplement, for details). Phosphorus content, which has been posited as a proxy for fossilized organic matter at vents [26], was also evaluated using this technique.

Measurements of the potential fossil microorganisms (hereafter referred to as ‘microfossils') were made from SEM images using the software ImageJ v. 1.46r [27]. Only microstructures with a distinctly circular or elliptical cross section, i.e. those likely to have a biogenic in origin, were measured. For statistical tests, diameter measurements from microfossils were divided into four data groups based on their location of occurrence. Shapiro–Wilk normality tests were used to determine if microfossil diameters were normally distributed, and *F*-tests to compare variances between data pairs. Two-sample Kolmogorov–Smirnov tests were subsequently used to compare the cumulative distributions of diameter measurements between data pairs. All three types of statistical test were performed in R [28].

Modern unmineralized and recently mineralized microbial filaments occurring on the tubes of the hydrothermal vent polychaete *Alvinella* sp. were imaged using SEM, for comparison with the Yaman Kasy microfossils. Unmineralized microorganisms were coated with 20 nm gold-palladium, while mineralized microbes were embedded in resin blocks, polished and coated with carbon as for the Yaman Kasy material.

Three-dimensional reconstructions of microfossils associated with both mineralized *Alvinella* and Yaman Kasy tubeworms were made through focused ion beam-SEM (FIB-SEM) tomography, using an FEI Quanta 3D FEG, at The NanoVision Centre, Queen Mary University of London, UK. Regions of interest within fossil tube walls prepared as polished blocks were selected using SEM, and were subsequently coated with platinum via ion beam induced chemical vapour deposition. Trenches were milled around these, after which the regions of interest were sequentially milled and imaged in approximately 14–20 nm thick slices. A total of 126–575 slices were generated for each region of interest. Slices were stacked, aligned and microfossils within these were segmented using Amira software (ThermoFisher Scientific).

## 3. Results

### (a) Occurrence of microfossils

**Figure 1.**
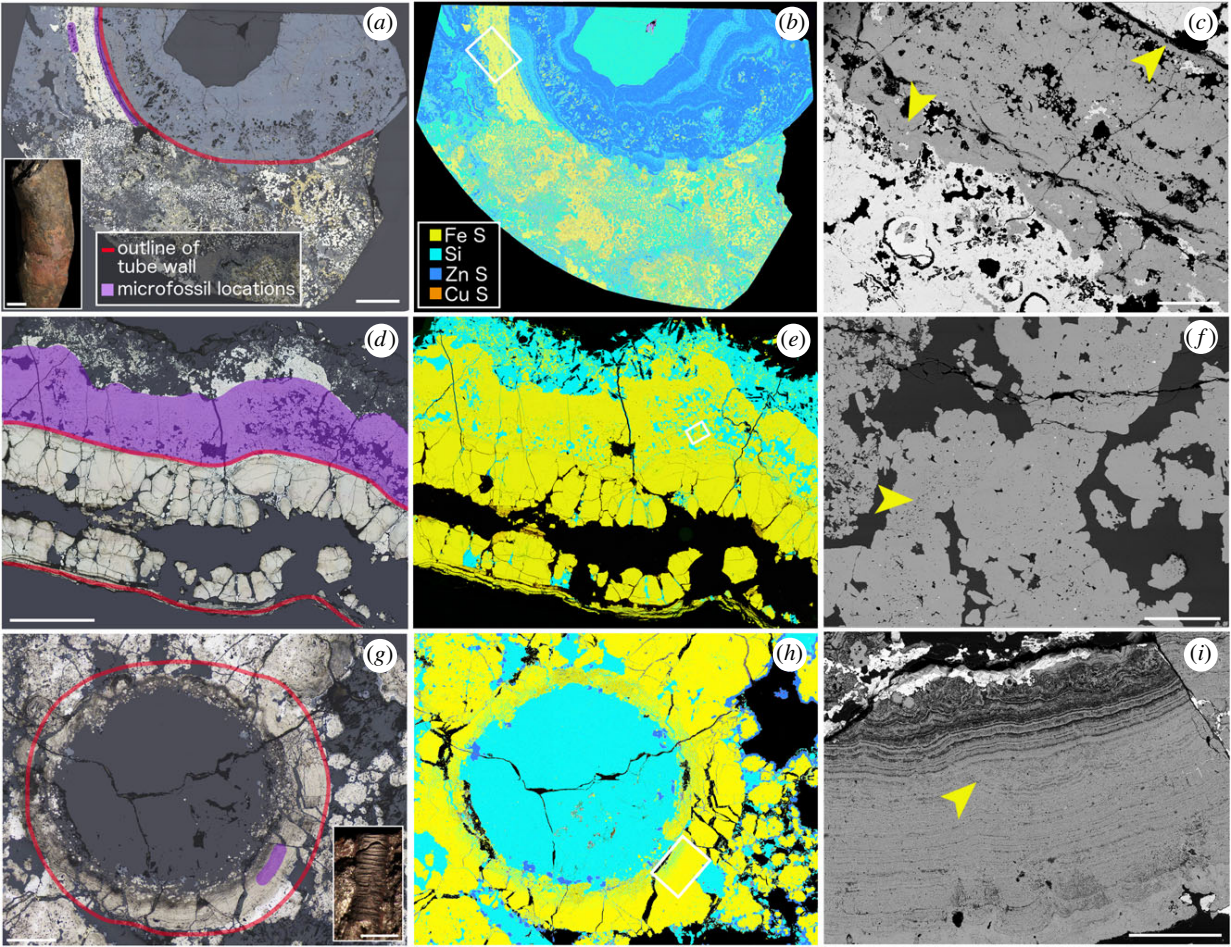
Sections of fossilized tubes from the Yaman Kasy deposit associated with microfossils. (*a*,*d*,*g*) reflected light images (tube rims and areas containing microfossils are highlighted), (*b*,*e*,*h*) elemental maps, (*c*,*f*,*i*) detail of areas where microfossils occur. (*a*) Transverse section of a *Yamankasia rifeia* tube (Yr_61633) in which only a section of colloform pyrite that formed on the outer tube surface has been preserved, scale is 3 mm; insert—left, an example of a *Y. rifeia* tube in hand specimen, scale bar is 10 mm; insert—right, key to colours in (*a*, *d* and *g*). (*b*) Elemental map of (*a*); *insert*, key to elemental maps (*b*, *e* and *h*). (*c*) SEM image of boxed area in (*b*) showing the colloform pyrite band in greater detail, and where microfossils occur within it. Small filaments occur along the inner surface of the band, and larger filaments are found along its outer edge (yellow arrows), scale bar is 500 µm. (*d*) Longitudinal section of an additional *Y. rifeia* tube (Yr_OR6468), in which microfossils occur in approximately 2 mm thick band of pyrite located on the outside of the fossilized tube, scale bar is 3 mm. (*e*) Elemental map of section in (*d*). (*f*) Detail of boxed area in (*e*) showing abundant microbial clumps in this region (yellow arrow), scale bar is 100 µm. (*g*) Transverse section of an *Eoalvinellodes annulatus* tube (Eo_YKB1), in which microfossils occur in a small area of colloform pyrite forming the tube wall, scale bar is 400 µm; insert, example of an *E. annulatus* tube in hand specimen, scale bar is 1 mm. (*h*) Elemental map of section in (*g*). (*i*) Detail of boxed area in (*h*) showing tube wall containing microfossils (yellow arrow), scale bar is 100 µm.

Microfossils were found in three sections of tubes from Yaman Kasy. They occurred in pyrite with colloform (or finely layered) growth, and fine-grained non-colloform pyrite. Tubes preserved by framboidal pyrite had no microfossils. Microfossils were found in a transverse and a longitudinal section of two separate *Y. rifeia* tubes (figure 1*a*–*f*) and a transverse section of an *E. annulatus* tube (figure 1*g–i*). In the *Y. rifeia* transverse section (Yr_61633; figure 1*a*–*c*), microfossils occurred within an approximately 2 mm thick layer of colloform pyrite that is considered to have grown on the exterior tube wall surface [20]. Within this layer, small microfossils occurred along the inner rim closest to where the original outer tube wall would have been, and slightly larger microfossils occurred towards the outer rim of the pyrite layer (figure 1*a*,*c*). Microfossils (figure 1*f*) in the *Y. rifeia* longitudinal section (Yr_OR6468; figure 1*d*) were found within an approximately 3 mm thick layer of fine-grained pyrite in which spaces were infilled by silica (figure 1*e*). This pyrite layer is also positioned on the outside of the fossil tube wall (figure 1*f*). In the *E. annulatus* tube transverse section (Eo_YKB1; figure 1*g*), microfossils occurred within a section of the fossil tube wall preserved as finely interlayered colloform pyrite and silica (figure 1*h*–*i*). Many of these microfossils were observed to crosscut the pyrite-silica banding.

### (b) Morphology and preservation of microfossils

**Figure 2.**
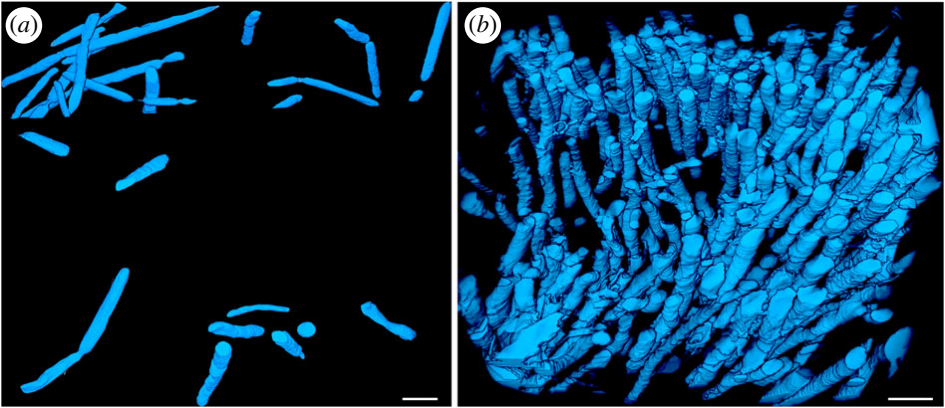
FIB-SEM reconstructions of microfossils. (*a*) Microfossils preserved within Yaman Kasy fossil specimen Yr_OR6468, scale bar is 2 µm; and (*b*) microfossils preserved within recently mineralized tube walls of *Alvinella* spp., scale bar is 2 µm. (Online version in colour.)

**Figure 3.**
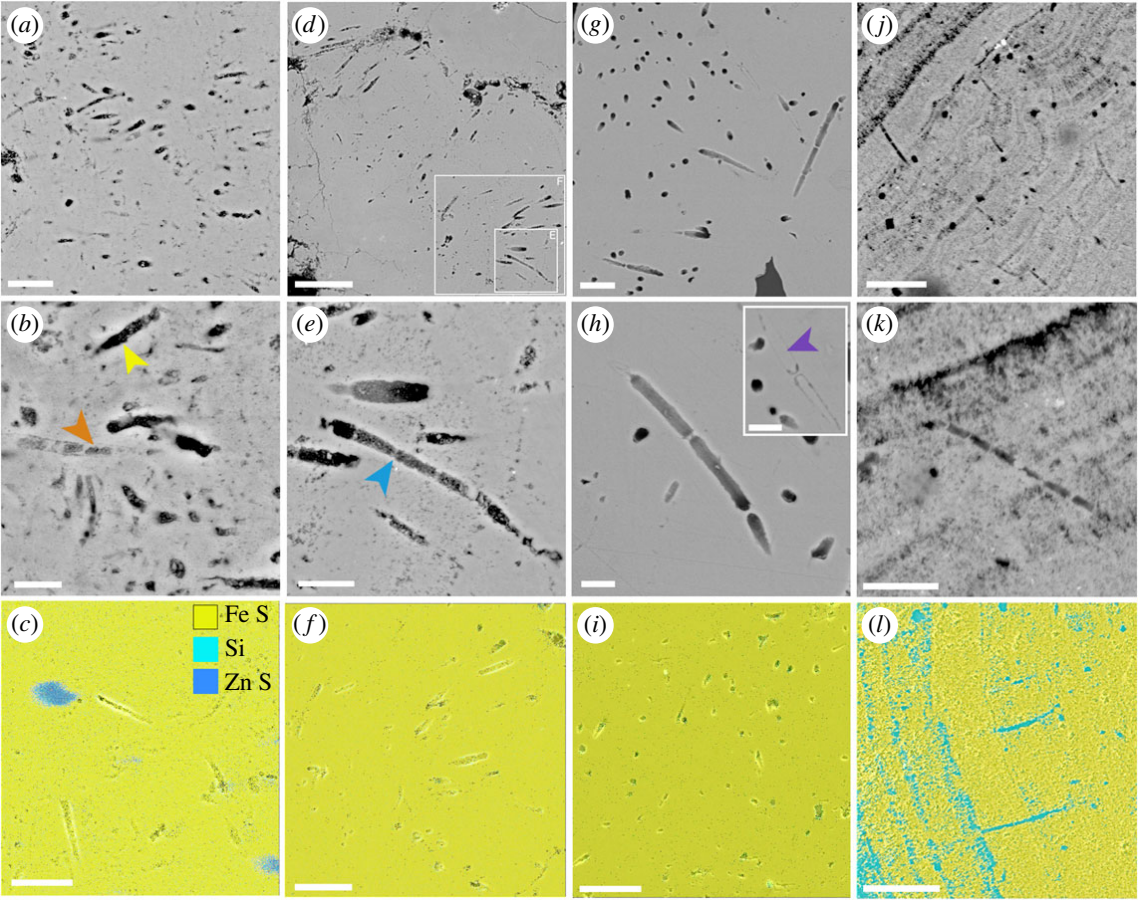
Morphology and elemental composition of microfossils associated with Yaman Kasy worm tube fossils. (*a*–*c*) Microfossils found along the inner rim of the colloform pyrite band within Yr_61633: (*a*) cluster of filamentous microfossils, scale bar is 5 µm; (*b*) detail of filamentous microfossils in (*a*), showing preservation of many hollow filaments (yellow arrow) as well as one which appears to retain a sheath (orange arrow), scale bar is 2 µm; and (*c*) elemental map of Yr_61633 inner pyrite rim microfossils, scale bar is 5 µm (*note* (*a*–*c*) are all of different areas). (*d*–*f*) Microfossils found along the outer rim of the colloform pyrite band within Yr_61633: (*d*) cluster of filamentous microfossils, scale bar is 20 µm (locations of (*e* and *f*) are shown); (*e*) detail of a microfossil with septae (blue arrow), scale bar is 4 µm; and (*f*) elemental map of Yr_61633 outer pyrite rim microfossils, scale bar is 10 µm. (*g*–*i*) Microfossils preserved in *Y. rifeia* longitudinal section (Yr_OR6468): (*g*) cluster of filamentous microfossils resembling chains of rods, scale bar is 5 µm; (*h*) detail of microfossils of the type pictured in (*g*), scale bar is 2 µm, insert, microfossil infilled by pyrite (purple arrow), scale bar is 2 µm; and (*i*) elemental map of Yr_OR6468 microfossils, scale bar is 10 µm (note (*g*–*i*) are all of different areas). (*j*–*l*) Microfossils preserved in *E. annulatus* transverse section (Eo_YKB1): (*j*) cluster of septate filamentous microfossils, scale bar is 10 µm; (*k*) detail of a septate microfossil from Eo_YKB1, scale bar is 5 µm; and (*l*) elemental map of Eo_YKB1 microfossils, scale bar is 10 µm (note (*j*–*l*) are all of different areas).

The Yaman Kasy microfossils occur in a variety of orientations, with FIB-SEM tomography three-dimensional reconstructions, and occasional microfossil longitudinal sections, revealing filamentous morphologies (figures 2*a* and 3). They generally formed clusters of filaments with a similar diameter, but their density within these clusters varied between the four locations where microfossils were found. (The four locations being: Yr_61633 inner rim of colloform pyrite (figure 3*a*–*c*); Yr_61633 outer rim of colloform pyrite (figure 3*d–f*); Yr_OR6468 (figure 3*g–i*); Eo_YKB1 (figure 3*j–l*); electronic supplementary material, figure S2.) The distributions of microfossil diameter measurements also varied between the four above locations (electronic supplementary material, figure S2), and were significantly different for all location data pairs (electronic supplementary material, tables S1–S3). Occasionally, filaments with visibly different diameters were preserved alongside each other, such as the orange-arrowed filament in figure 3*b*, and the adjacent smaller, vertically oriented filaments.

The filamentous microfossils were often curved (figure 3*a–e*,*h–j*), and a subset was cross-cut by transverse septae that occurred at regular intervals (figure 3*b*,*e*,*h*,*k*). Distances between septae (in relation to filament diameter) were visibly greater for microfossils in Yr_OR6468 than in Yr_61633 and Eo_YKB1. In addition, individual Yr_OR6468 ‘cells' (figure 3*g–h*) had a rod-like appearance, whereas the endings of ‘cells' of septate microfossil filaments from Yr_61633 and Eo_YKB1 showed a large area of attachment to one another. A microfossil filament in figure 3*b* (arrowed) appears to be sheathed, owing to the encasement of cell-like bodies within a tubular structure.

Detailed EDS maps of the Yaman Kasy microfossils show that they are mainly preserved by iron sulfides (figure 3*c*,*f*,*i*,*l*; confirmed to be pyrite using reflected light microscopy) as hollow moulds delineated by pyrite, with silica infilling cells within one of the specimens (figure 3*l*). Occasionally, the microfossils were also infilled by pyrite (figure 3*b*,*h* insert), and septae were also formed of pyrite. There appeared to be no clear variation in pyrite composition of sample areas containing microfossils, and those that did not. Phosphorus was not detected around microfossils in either Yr_61633 or Yr_OR6468 by EPMA (electronic supplementary material, figures S3–S6 and tables S4–S5).

## 4. Interpretations and discussion

The microfossils found in association with fossil tubes from Yaman Kasy meet many of the suggested criteria for genuine microbial fossils [29]. Importantly, they occur within an appropriate context, as a diverse range of microorganisms are often found growing on the surfaces of annelid tubes and vent chimney sulfides in modern vent environments [30–32]. All of the Yaman Kasy microfossils occur as a population of filaments of a similar size and morphology, which are often clustered together (figure 3*a*,*d*,*g*) and thus resemble clumps of modern vent microbial filaments [19]. The small filaments within sample Yr_61633 have a mat-like appearance, as they are distributed within a very narrow zone of pyrite that is thought to have grown directly onto the outer tube wall [20]. The microfossils themselves have tubular morphologies with mostly constant diameters. The curvature of the filaments suggests that they were originally flexible, and had mostly hollow interiors apart from where infilled by silica or pyrite. In addition, the septate divisions within many of the filaments delineate spaces indicative of cells. The observed microfossil textures are not the result of microbial leaching of pyrite [33,34] as they occur throughout the pyrite matrix in which they are preserved, and are present in a range of orientations (figure 2*a*).

**Figure 4.**
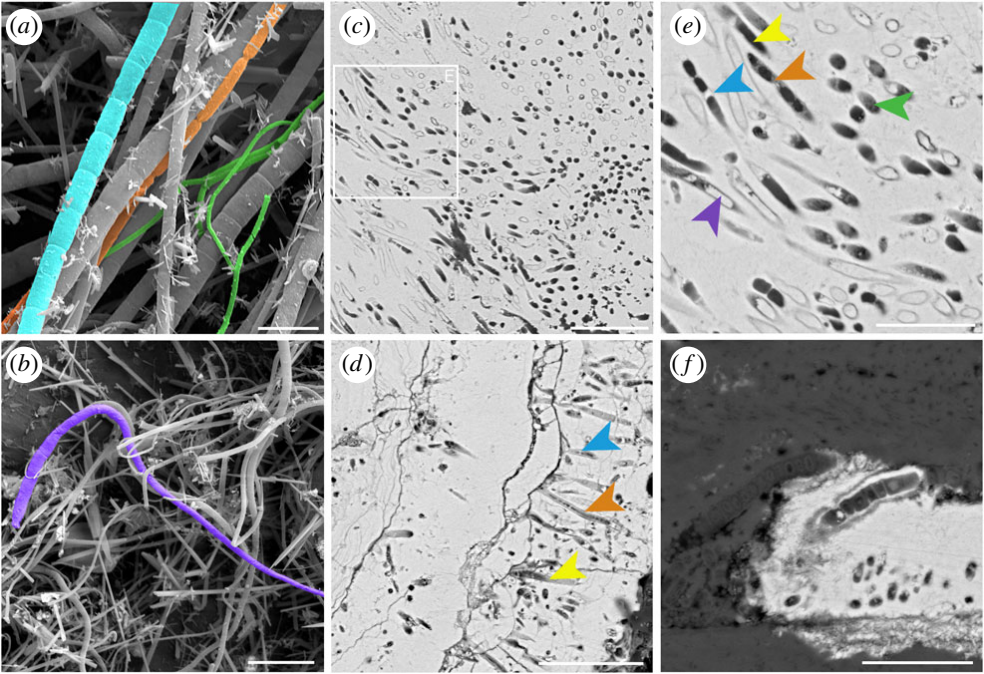
Microorganisms and microbial fossils associated with the tubes of the hydrothermal vent annelid *Alvinella* sp. (*a*,*b*) Unmineralized filamentous microorganisms from the inside surface of an *Alvinella* sp. tube exhibiting a variety of morphologies, including non-septate filaments (green), septate filaments (blue), ‘chain-of-rods’-type filament (orange) and a tapering filament (purple). Scale bars are 5 µm in (*a*) and 10 µm in (*b*). (*c*–*f*) Microorganisms mineralized alongside *Alvinella* sp. tubes: (*c*) clump of filaments in a variety of orientations, scale bar is 10 µm; (*d*) filaments arranged longitudinally within a band of pyrite thereby demonstrating mat-like growth, scale bar is 10 µm (some of the filaments appear hollow (yellow arrow), whereas others exhibit septae (blue arrow) as well as replacement of a microbial sheath by pyrite (orange arrow)); (*e*) detail of area in (*c*) showing hollow filaments (yellow arrow), filaments with septae formed of pyrite (blue arrow), septae and sheath formed of silica (orange arrow), as well as filaments infilled by pyrite (purple arrow) and others infilled by silica (green arrow), scale bar is 5 µm; and (*f*) filamentous microorganisms preserved in exceptional detail by both pyrite and silica (silica—dark grey, pyrite—light grey), that reveals sub-cellular aspects, scale bar is 5 µm.

The interpretation of the Yaman Kasy microfossils as fossilized microorganisms is further supported by both their very close resemblance to modern day hydrothermal vent filamentous microorganisms (figures 2*b* and 4), and studies which have shown that microorganisms as small as 1 µm in diameter can be fossilized with high fidelity by pyrite at vents [19]. Like the Yaman Kasy microfossils, modern vent microbial filaments can be tapered as well as septate [31,35,36]. For example, septate, non-septate, chain-of-rods (figure 4*a*) and tapering (figure 4*b*) microbial morphologies can all be observed on the surfaces of *Alvinella* tubes. Following mineralization, the clustering (figures 3*a* and 4*c*) and mat-like (figure 4*d*) growth habits of these microorganisms are maintained. At modern vents, pyrite and silica also preserve fine details such as septae, microbial sheaths and cell contents (figure 4*e–f*), and occasionally infill microfossils preserved as moulds (figure 4*e–f*; electronic supplementary material, figure S7). While phosphorus was not detected around the Yaman Kasy microfossils (electronic supplementary material, figures S3–S6; tables S4–S5), the retention of this element during the mineralization of organic matter at modern vents (Maginn *et al.* [26]) may be specific to *Alvinella* tubes and their particular microbial community.

**Figure 5.**
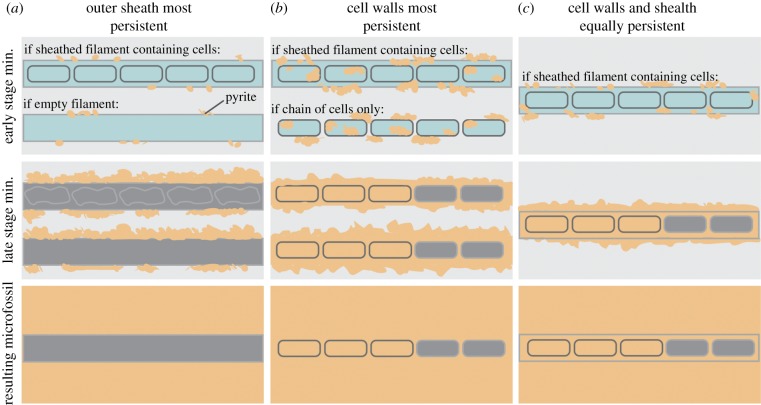
Fossilization models for hydrothermal vent microorganisms. In scenario (*a*), the resulting microfossil is an empty filament moulded of pyrite. There could be two starting microorganism types for this: a filament containing cells in which the cells are not preserved, and a filament that does not contain cells. With both of these starting filaments, mineralization would need to be confined to mainly the outer wall, while any contents degrade within the pyrite tomb. Under scenario (*b*), the resulting microfossil looks like a chain of cells. Either these cells never had a sheath, or such a sheath was not preserved. Mineralization probably propagated along the cell walls, with the cell volume also being infilled in some cases. The microfossil that results in scenario (*c*) shows preservation of both microbial cells and an outer sheath, therefore the starting filament must have contained both of these features. Both the cell walls and sheath walls probably had equal persistence, and cell volumes may also be infilled as these filaments are mineralizing.

Based on the observed Yaman Kasy microfossil morphologies, there are a number of preservational pathways and original microbial growth-types from which the microfossils could have resulted (figure 5). Bacteria are known to concentrate minerals along with their surfaces [37,38] and may also induce mineralization at vents [39,40]. Thus, the range of preservations observed in ancient and recently mineralized microorganisms may also be linked to preferential mineral accumulations within various parts of the microbial filaments/cells. Empty filamentous microfossil pyrite moulds (e.g. figure 2*b*) may have either formed from filaments that contained no cells, or if cells were present, mineralization may have been concentrated along the outer sheath walls, thus preventing mineralization of inner cells (figure 5*a*). For microfossils that exhibit septate divisions but no sheath (figure 2*e*,*h*,*k*), there may have originally been a sheath that was completely replaced by pyrite, while mineralization appears to have been concentrated along the cell walls (figure 5*b*). Infilling of cells was probably contemporaneous with cell wall mineralization [39], while the empty nature of some cells could have resulted from their cell walls mineralizing before vent fluids were able to penetrate into the cell interior. Microfossils that demonstrate preservation of both cells and sheaths (figure 2*b*) indicate that the sheath and cell walls had similar resistance and were probably mineralized at the same time (figure 5*c*). Microbial sheaths were only well preserved in a subset of recently mineralized vent microorganisms (figure 4*d–f*), suggesting that this type of preservation may be less common. While the preservation of microfossils also appears to not affect pyrite composition at EPMA detection scale (electronic supplementary material, figures S3–S6 and tables S4–S5), finer scale analyses that target pyrite directly delineating microfossils could shed insights into any effects of microbial presence on mineral precipitation.

Microorganisms from modern vent environments are largely described using molecular and not morphological methods (e.g. Schrenk *et al*., [41]; Anderson *et al*., [42]), making it difficult to determine the probable taxonomic identity of the Yaman Kasy microfossils. Microorganisms can also exhibit convergent morphologies, and microbial morphologies may vary in relation to environmental conditions [43]. Archaea, *ɛ*-Proteobacteria and *Aquificales* have all been identified as prominent members of modern vent microbial communities [1,2], and while bacteria are more likely to occur as filaments, Archaea can occasionally also take this form [44]. Nevertheless, the Yaman Kasy microfossils are perhaps more likely those of bacteria, as bacteria are more abundant in modern vents and often form mats of filaments in this environment.

The occurrence of both ‘chain-of-rods' microfossils (figure 3) with curved endings resembling a *Streptobaccillus*-type morphology, and microfossils resembling microbial trichomes [45] (figure 3*b*,*e*,*k*), as well as mixing of different sizes of filaments (figure 3*b*), alludes to a diversity of different microorganisms associated with the Yaman Kasy tubes. The original microbial diversity of the Yaman Kasy palaeocommunity was undoubtedly greater than the microfossils described here suggest, as microbial morphologies such as coccoids that are observed to occur within modern vent environments [3,46] were probably not fossilized. In addition to their morphologies, Yaman Kasy microfossils from the four locations where they were observed (figure 1*c*,*f*,*i*) exhibit different diameter distributions as well as variations in microfossil density. This reflects the characteristics of microbial mats within modern day hydrothermal vent environments, which are often diverse and exhibit spatial variation in the degree to which microorganisms of a particular type are mixed in with other sizes and morphotypes of microorganisms [2] (figure 4*a,b*). This results from the wide range of niches available at vents, and this study demonstrates, to our knowledge, the first evidence that microorganisms, in association with large metazoan animals, were taking advantage of the assortment of niches available at vents approximately 440 Ma.

The locations of a subset of the Yaman Kasy microfossils (figure 1*a*,*e*) suggest that microorganisms were living on and around metazoan tube surfaces and were fossilized alongside the tubes, very similar to the preservation of annelid tubes and their epiphytic microorganisms recently observed within modern vent environments [19]. This preservation demonstrates that associations between microorganisms and animals that have been observed within modern vents, such as commensalism and episymbioses [32,47,48], may also be detected within the fossil record of ancient vent communities. With regard to the palaeoecology of the Yaman Kasay vent, our data confirm suggestions that the large tubeworms associated with this vent may have been living in close proximity to high-temperature venting [20], given the clear similarities of the microbial associations with modern alvinellid tubeworms. This implies that adaptation to the highest-temperature part of the vent habitat occurred soon after metazoans were first able to adapt to the vent conditions. For some of the resulting microfossils, such as those for which pyrite may preserve cellular details, it may also be possible to gain a good understanding of what the original microorganisms looked like (figure 5). These results also show that microbial colonization of metazoan tubes is an association that has a very long fossil history, stretching back to the earliest known hydrothermal vent community, and demonstrates the potential for detailed microbial preservation within even older hydrothermal vent deposits.

## Supplementary Material

Electronic Supplementary Material: methods, figures and tables

## Data accessibility

Supplementary methods, figures and data tables to this manuscript can be found in the electronic supplementary material. Data used to generate figure 2 is available via the Dryad Digital Repository: http://dx.doi.org/10.5061/dryad.8171kv1 [49].
