# Supplementary material for: Microbial-tubeworm associations in a 440 million year old hydrothermal vent community
Source: Proc Biol Sci. 2018 Nov 14;285(1891):20182004. doi: 10.1098/rspb.2018.2004 (PMC6253371; doi:10.1098/rspb.2018.2004)
Supplement: Electronic Supplementary Material: methods, figures and tables [file rspb20182004supp1.docx]

Electronic Supplementary Material:

**Microbial-tubeworm associations in a 440 million year old hydrothermal vent community.**

Proceedings of the Royal Society B, 20182004. http://dx.doi.org/10.1098/rspb.2018.2004

Magdalena N. Georgieva

Crispin T. S. Little

Russell J. Bailey

Alexander D. Ball

Adrian G. Glover

**Methods Supplement: Electron probe microanalysis**

Previous studies of the mineralisation of polychaete tubes at hydrothermal vents indicate that elevated levels of phosphorus may be associated with mineralised organic matter (Maginn *et al.*, 2002). Pyrite content and phosphorus occurrence around Yaman Kasy microstructures was assessed using wavelength-dispersive spectrometry (WDS) point analyses, within a Cameca SX-100 electron microprobe (EPMA; Natural History Museum UK). The instrument was operated using an accelerating voltage of 20 kV and a probe current of 20 nA, and locations of data points are shown in Figures S2 to S5. These analyses showed that in all of the data points, phosphorus was either not present or present in amounts below the detection limits of the instrument (Tables S1 to S2).


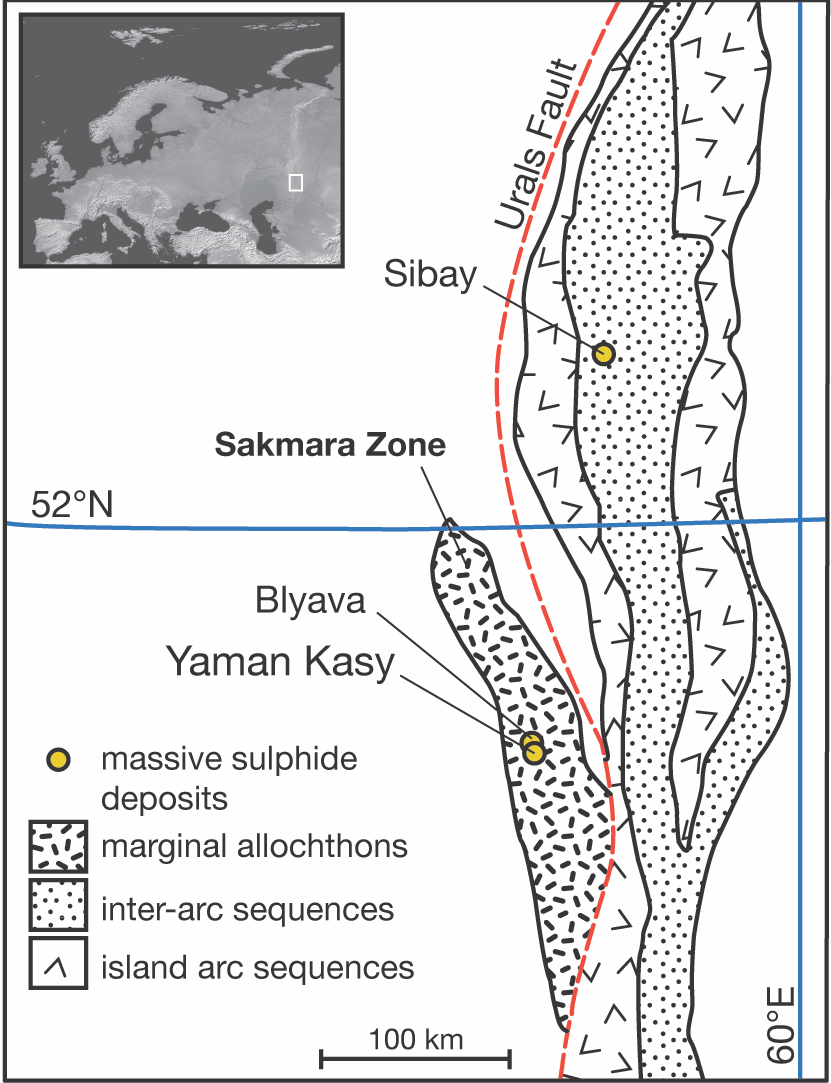


**Figure S1** Location of Yaman Kasy and other nearby massive sulphide deposits.

Inset shows the location of the enlarged map area. Map adapted from Little *et al.* (1999).

**Table S1** Results of Shapiro-Wilk normality tests of microstructure diameter distributions.

Non-significant results are highlighted, TS - transverse section, LS - longitudinal section.

|  | N | W | p-value |
| --- | --- | --- | --- |
| Yr_61633 inner | 919 | 0.9665 | 1.069e-13 |
| Yr_61633 outer | 131 | 0.9837 | 0.1183 |
| Yr_OR6468 | 397 | 0.9869 | 0.001239 |
| Eo_YKB1 | 359 | 0.9152 | 2.437e-13 |

**Table S2** Results of *F*-tests for the comparison of variance between dataset pairs of microstructure diameter measurements.

Non-significant results are highlighted.

|  | Yr_61633 inner | Yr_61633 outer | Yr_OR6468 |
| --- | --- | --- | --- |
| Yr_61633 outer | F = 0.2072, num df = 918, denom df = 130, p-value < 2.2e-16 |  |  |
| Yr_OR6468 | F = 0.8954, num df = 918, denom df = 396, p-value = 0.1872 | F = 4.3224, num df = 130, denom df = 396, p-value < 2.2e-16 |  |
| Eo_YKB1 | F = 0.6952, num df = 918, denom df = 358, p-value = 2.248e-05 | F = 3.356, num df = 130, denom df = 358, p-value < 2.2e-16 | F = 0.7764, num df = 396, denom df = 358, p-value = 0.01406 |

**Table S3** Results of Kolmogorov-Smirnov tests for comparison of microstructure diameter distributions between dataset pairs.

|  | Yr_61633 inner | Yr_61633 outer | Yr_OR6468 |
| --- | --- | --- | --- |
| Yr_61633 outer | D = 0.7438, p-value < 2.2e-16 |  |  |
| Yr_OR6468 | D = 0.5445, p-value < 2.2e-16 | D = 0.634, p-value < 2.2e-16 |  |
| Eo_YKB1 | D = 0.2737, p-value < 2.2e-16 | D = 0.6477, p-value < 2.2e-16 | D = 0.347, p-value < 2.2e-16 |

**
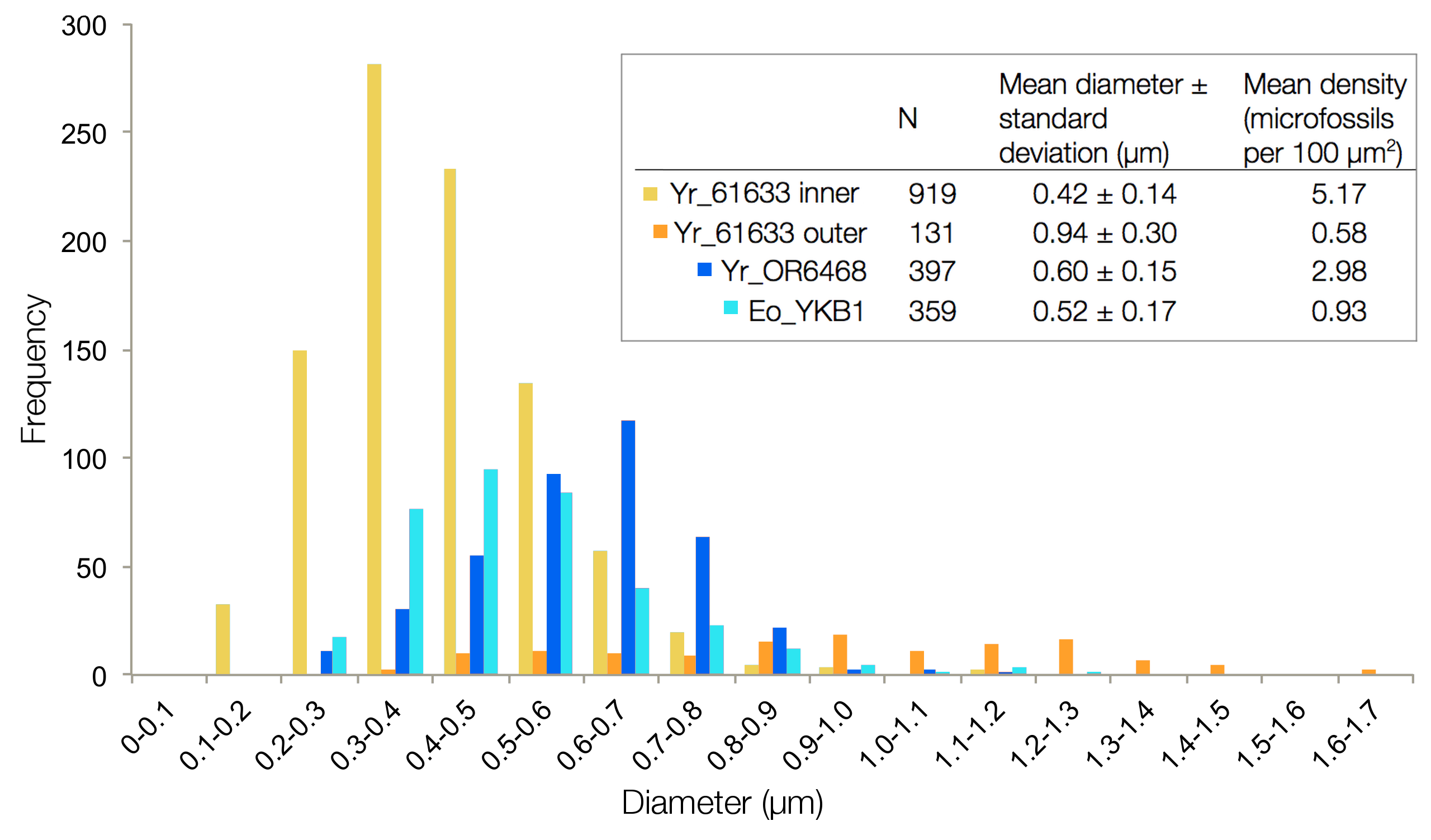
**

**Figure S2** Diameter distributions and density measurements for Yaman Kasy microfossils.

Bars are coloured according to the four around-tube locations where microfossils were found (Yr_61633 inner rim of colloform pyrite; Yr_61633 outer rim of colloform pyrite; Yr_OR6468; Eo_YKB1).

**Figure S3** Location of EPMA data points within Area 1, sample Yr_61633.

**Figure S4** Location of EPMA data points within Area 2, sample Yr_61633.

**Figure S5** Location of EPMA data points within Area 3, sample Yr_OR6468.

**Figure S6** Location of EPMA data points within Area 4, sample Yr_OR6468.


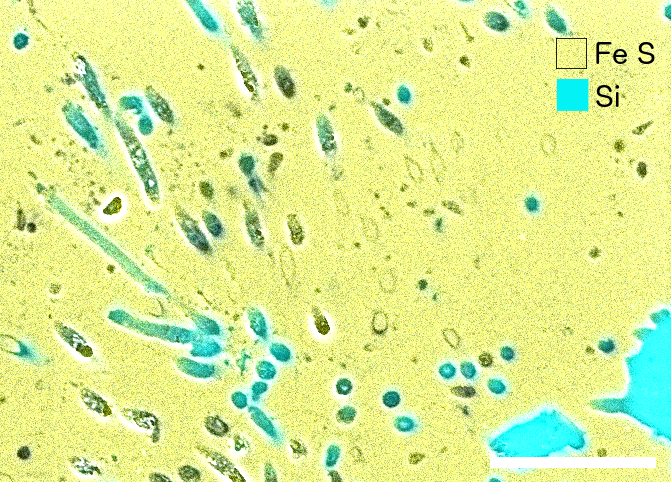


**Figure S7** Elemental composition of mineralised microbial filaments preserved alongside the tubes of *Alvinella* sp.

Scale bar is 7 µm.

**Table S4** Pyrite composition (in weight %) from EPMA analyses of pyrite around Yaman Kasy microfossils and in adjacent pyrite that does not contain them (grey rows).

| Figure S3 (Area 1, Yr_61633) | | | |  |  |  |  |  |  |  |  |  |  |  |  |  |  |  |
| --- | --- | --- | --- | --- | --- | --- | --- | --- | --- | --- | --- | --- | --- | --- | --- | --- | --- | --- |
| Point | P | V | Mn | Fe | Co | Ni | Cu | Zn | Si | S | Cr | Ti | Ca | Mg | As | Sr | Ba | Pb |
| 1 / 1. | 0.00 | 0.00 | 0.03 | 44.45 | -0.01 | 0.00 | 0.09 | 0.12 | 0.01 | 52.58 | 0.00 | 0.00 | 0.00 | 0.01 | 0.15 | -0.05 | 0.00 | 0.54 |
| 1 / 2 . | 0.01 | 0.00 | 0.02 | 44.33 | -0.02 | 0.00 | 0.10 | 0.54 | 0.00 | 52.04 | 0.01 | 0.00 | -0.01 | 0.01 | 0.08 | -0.02 | 0.00 | 0.51 |
| 1 / 3 . | 0.00 | 0.01 | 0.02 | 44.31 | -0.01 | 0.00 | 0.15 | 0.14 | 0.00 | 51.94 | 0.00 | 0.00 | 0.00 | 0.00 | 0.18 | -0.04 | 0.02 | 0.56 |
| 1 / 4 . | 0.01 | -0.01 | 0.03 | 44.30 | 0.03 | 0.00 | 0.09 | 0.22 | 0.00 | 51.59 | -0.01 | 0.00 | -0.01 | 0.01 | 0.08 | -0.03 | 0.01 | 0.63 |
| 1 / 5 . | 0.01 | 0.00 | 0.03 | 44.06 | -0.02 | 0.00 | 0.09 | 0.12 | 0.00 | 51.27 | 0.00 | -0.01 | 0.00 | 0.00 | 0.16 | -0.02 | 0.01 | 0.65 |
| 1 / 6 . | 0.01 | 0.00 | 0.03 | 43.12 | -0.02 | 0.01 | 0.18 | 0.24 | 0.03 | 48.17 | 0.01 | -0.01 | 0.02 | -0.02 | 0.51 | -0.09 | -0.02 | 0.69 |
| 1 / 7 . | 0.03 | 0.00 | 0.02 | 42.86 | 0.02 | 0.00 | 0.18 | 0.52 | 0.07 | 45.56 | -0.02 | -0.01 | 0.07 | -0.01 | 0.70 | -0.24 | -0.01 | 0.77 |
| 1 / 8 . | -0.01 | 0.01 | 0.03 | 43.69 | 0.01 | 0.00 | 0.16 | 0.23 | 0.01 | 51.25 | 0.01 | 0.00 | 0.01 | -0.01 | 0.58 | -0.07 | -0.01 | 0.44 |
| 1 / 9 . | 0.01 | 0.00 | 0.03 | 43.60 | 0.01 | 0.00 | 0.19 | 0.20 | 0.02 | 50.50 | 0.01 | 0.00 | 0.01 | -0.01 | 0.59 | -0.08 | 0.00 | 0.45 |
| 1 / 10 . | 0.00 | 0.00 | 0.03 | 44.42 | 0.00 | -0.02 | 0.09 | 0.16 | 0.01 | 51.79 | -0.01 | -0.01 | 0.00 | 0.00 | 0.19 | -0.02 | 0.01 | 0.56 |
| 1 / 11 . | 0.00 | 0.00 | 0.02 | 43.85 | -0.01 | -0.01 | 0.17 | 0.27 | 0.01 | 49.71 | -0.01 | 0.00 | 0.00 | -0.01 | 0.55 | -0.07 | 0.00 | 0.37 |
| 1 / 12 . | 0.00 | 0.00 | 0.02 | 44.13 | 0.01 | -0.01 | 0.14 | 0.17 | 0.01 | 51.15 | 0.01 | -0.01 | 0.00 | 0.00 | 0.13 | -0.05 | 0.01 | 0.59 |
| 1 / 13 . | 0.00 | 0.00 | 0.02 | 44.42 | -0.01 | 0.00 | 0.11 | 0.11 | 0.00 | 51.80 | -0.02 | 0.00 | -0.01 | 0.01 | 0.18 | -0.02 | -0.03 | 0.55 |
| 1 / 14 . | 0.00 | 0.00 | 0.01 | 43.70 | 0.01 | 0.01 | 0.17 | 0.42 | 0.03 | 47.69 | -0.01 | 0.00 | 0.05 | -0.01 | 0.65 | -0.09 | -0.01 | 0.58 |
| 1 / 15 . | 0.01 | 0.00 | 0.04 | 43.57 | 0.01 | 0.00 | 0.14 | 0.25 | 0.02 | 50.02 | 0.00 | 0.01 | 0.04 | -0.01 | 0.41 | -0.07 | 0.01 | 0.55 |
| 1 / 16 . | 0.00 | 0.00 | 0.03 | 43.84 | 0.01 | 0.00 | 0.21 | 0.29 | 0.02 | 50.55 | 0.00 | -0.01 | 0.04 | -0.01 | 0.47 | -0.09 | 0.00 | 0.57 |
| 1 / 17 . | 0.00 | -0.01 | 0.04 | 44.34 | -0.01 | 0.00 | 0.10 | 0.20 | 0.00 | 51.57 | -0.01 | -0.02 | 0.00 | 0.00 | 0.18 | -0.02 | -0.01 | 0.69 |
| 1 / 18 . | 0.01 | 0.00 | 0.05 | 44.12 | 0.00 | 0.01 | 0.13 | 0.30 | 0.01 | 51.61 | 0.00 | 0.00 | 0.01 | 0.01 | 0.29 | -0.05 | -0.01 | 0.57 |
| 1 / 19 . | 0.00 | 0.00 | 0.02 | 44.55 | -0.02 | 0.00 | 0.10 | 0.23 | 0.00 | 52.01 | 0.00 | 0.00 | 0.00 | 0.00 | 0.14 | -0.02 | 0.00 | 0.46 |
| 1 / 20 . | 0.00 | 0.00 | 0.05 | 44.25 | 0.00 | 0.00 | 0.21 | 0.31 | 0.01 | 51.91 | 0.01 | 0.00 | 0.00 | 0.00 | 0.28 | -0.04 | -0.01 | 0.55 |
| 1 / 21 . | 0.01 | 0.00 | 0.03 | 44.19 | 0.00 | 0.01 | 0.18 | 0.28 | 0.02 | 51.23 | 0.00 | 0.00 | 0.01 | 0.01 | 0.23 | -0.08 | 0.02 | 0.60 |
| 1 / 22 . | 0.01 | 0.00 | 0.04 | 44.01 | -0.01 | 0.00 | 0.09 | 0.74 | 0.00 | 52.13 | 0.00 | -0.01 | 0.00 | 0.00 | 0.13 | -0.04 | -0.01 | 0.55 |
| 1 / 23 . | 0.00 | 0.00 | 0.03 | 43.25 | 0.01 | -0.02 | 0.13 | 2.08 | 0.00 | 51.05 | 0.00 | 0.00 | 0.00 | 0.00 | 0.12 | -0.01 | 0.00 | 0.60 |
| 1 / 24 . | 0.00 | -0.01 | 0.08 | 44.02 | 0.01 | 0.00 | 0.12 | 0.46 | 0.01 | 51.68 | 0.02 | 0.00 | 0.00 | 0.00 | 0.27 | -0.04 | 0.00 | 0.53 |
| 1 / 25 . | 0.00 | 0.00 | 0.04 | 43.81 | 0.00 | 0.00 | 0.12 | 0.46 | 0.02 | 51.00 | 0.01 | 0.05 | 0.00 | 0.01 | 0.29 | -0.08 | -0.02 | 0.54 |
| 1 / 26 . | 0.00 | 0.00 | 0.04 | 44.44 | 0.00 | 0.00 | 0.10 | 0.14 | 0.03 | 52.39 | 0.01 | 0.00 | 0.00 | 0.01 | 0.28 | -0.11 | -0.01 | 0.45 |
| 1 / 27 . | 0.00 | -0.01 | 0.03 | 44.46 | 0.01 | 0.01 | 0.11 | 0.25 | 0.10 | 52.04 | 0.02 | 0.01 | 0.00 | 0.00 | 0.13 | -0.37 | -0.01 | 0.50 |
| 1 / 28 . | 0.00 | 0.00 | 0.05 | 44.72 | 0.00 | 0.01 | 0.07 | 0.50 | 0.00 | 52.40 | -0.02 | -0.01 | 0.00 | 0.01 | 0.08 | -0.03 | 0.02 | 0.57 |
| 1 / 29 . | 0.01 | 0.00 | 0.08 | 44.30 | 0.01 | -0.01 | 0.08 | 0.43 | 0.02 | 50.62 | 0.00 | 0.00 | 0.00 | -0.02 | 0.70 | -0.07 | -0.01 | 0.42 |
| 1 / 30 . | 0.01 | 0.00 | 0.03 | 44.31 | 0.00 | -0.01 | 0.09 | 0.81 | 0.00 | 52.18 | -0.01 | 0.00 | 0.00 | 0.00 | 0.09 | -0.03 | 0.00 | 0.69 |
| Figure S4 (Area 2, Yr_61633) | | | |  |  |  |  |  |  |  |  |  |  |  |  |  |  |  |
| Point | P | V | Mn | Fe | Co | Ni | Cu | Zn | Si | S | Cr | Ti | Ca | Mg | As | Sr | Ba | Pb |
| 1 / 1 . | 0.00 | 0.00 | 0.01 | 43.09 | 0.01 | 0.00 | 0.10 | 1.01 | 0.01 | 50.69 | -0.01 | -0.01 | 0.00 | -0.01 | 0.57 | -0.04 | 0.02 | 0.42 |
| 1 / 2 . | 0.00 | 0.00 | 0.01 | 43.58 | 0.01 | 0.00 | 0.09 | 0.31 | 0.00 | 51.03 | 0.00 | 0.00 | -0.01 | 0.00 | 0.34 | -0.03 | -0.01 | 0.67 |
| 1 / 3 . | 0.00 | 0.00 | 0.02 | 43.34 | -0.01 | 0.00 | 0.11 | 0.53 | 0.01 | 50.55 | 0.00 | -0.01 | 0.00 | -0.02 | 0.72 | -0.01 | 0.01 | 0.44 |
| 1 / 4 . | -0.01 | 0.00 | 0.03 | 43.93 | 0.02 | 0.00 | 0.06 | 0.39 | 0.00 | 50.79 | 0.00 | 0.00 | 0.00 | -0.01 | 0.29 | -0.07 | 0.00 | 0.57 |
| 1 / 5 . | 0.01 | 0.00 | 0.02 | 44.00 | 0.00 | 0.02 | 0.07 | 0.23 | 0.00 | 51.52 | -0.01 | -0.01 | 0.00 | 0.00 | 0.18 | -0.02 | -0.01 | 0.60 |
| 1 / 6 . | 0.01 | 0.00 | 0.01 | 43.85 | -0.01 | 0.00 | 0.07 | 0.29 | 0.01 | 51.93 | -0.01 | 0.00 | 0.00 | 0.01 | 0.22 | -0.05 | 0.00 | 0.54 |
| 1 / 7 . | 0.01 | 0.00 | 0.02 | 43.91 | -0.01 | 0.00 | 0.09 | 0.39 | 0.05 | 51.24 | 0.00 | 0.00 | 0.00 | 0.03 | 0.25 | -0.13 | 0.00 | 0.30 |
| 1 / 8 . | 0.01 | 0.00 | 0.04 | 42.89 | 0.01 | 0.01 | 0.09 | 0.34 | 0.03 | 49.55 | -0.01 | 0.00 | 0.00 | -0.02 | 0.85 | -0.08 | 0.02 | 0.47 |
| 1 / 9 . | 0.00 | 0.01 | 0.02 | 43.62 | 0.00 | 0.00 | 0.10 | 0.42 | 0.01 | 50.39 | 0.01 | -0.01 | 0.01 | -0.01 | 0.41 | -0.07 | -0.01 | 0.39 |
| 1 / 10 . | 0.00 | -0.01 | 0.02 | 42.93 | 0.01 | 0.01 | 0.11 | 0.40 | 0.03 | 49.51 | 0.00 | 0.00 | 0.00 | -0.02 | 0.83 | -0.09 | -0.02 | 0.49 |
| 1 / 11 . | 0.00 | 0.00 | 0.04 | 42.85 | 0.01 | 0.01 | 0.10 | 0.48 | 0.00 | 50.32 | 0.01 | 0.01 | 0.00 | -0.03 | 0.84 | -0.02 | 0.00 | 0.51 |
| 1 / 12 . | 0.00 | 0.00 | 0.03 | 43.77 | 0.01 | 0.00 | 0.07 | 0.35 | 0.01 | 51.52 | 0.00 | -0.01 | 0.00 | 0.00 | 0.39 | -0.03 | -0.01 | 0.40 |
| 1 / 13 . | 0.00 | 0.00 | 0.03 | 43.55 | 0.00 | 0.01 | 0.09 | 0.47 | 0.01 | 51.53 | -0.01 | 0.00 | -0.01 | -0.01 | 0.41 | -0.03 | -0.01 | 0.31 |
| 1 / 14 . | 0.00 | 0.00 | 0.03 | 43.55 | 0.01 | -0.01 | 0.10 | 0.72 | 0.00 | 51.02 | -0.01 | 0.00 | 0.00 | 0.00 | 0.51 | -0.05 | 0.00 | 0.32 |
| 1 / 15 . | 0.00 | 0.00 | 0.04 | 43.34 | -0.01 | 0.00 | 0.06 | 0.38 | 0.01 | 50.69 | 0.02 | 0.00 | 0.00 | -0.01 | 0.48 | -0.07 | 0.02 | 0.30 |
| 1 / 16 . | 0.00 | -0.01 | 0.03 | 43.28 | 0.01 | 0.00 | 0.08 | 0.39 | 0.01 | 50.77 | 0.00 | 0.00 | 0.00 | -0.01 | 0.59 | -0.05 | 0.00 | 0.45 |
| 1 / 17 . | 0.01 | 0.00 | 0.05 | 43.10 | 0.01 | 0.01 | 0.11 | 0.48 | 0.02 | 49.81 | 0.01 | -0.01 | 0.01 | -0.01 | 0.72 | -0.06 | -0.01 | 0.40 |
| 1 / 18 . | 0.00 | 0.01 | 0.05 | 43.81 | 0.00 | 0.01 | 0.10 | 0.33 | 0.00 | 51.30 | 0.00 | 0.00 | 0.00 | 0.00 | 0.29 | -0.05 | 0.00 | 0.60 |
| 1 / 19 . | -0.01 | 0.00 | 0.03 | 43.88 | 0.01 | 0.01 | 0.06 | 0.21 | 0.06 | 51.60 | -0.01 | 0.00 | 0.00 | 0.01 | 0.18 | -0.07 | 0.00 | 0.54 |
| 1 / 20 . | 0.00 | 0.00 | 0.02 | 43.94 | -0.01 | -0.01 | 0.08 | 0.30 | 0.01 | 51.61 | 0.01 | -0.01 | 0.00 | 0.00 | 0.21 | -0.04 | 0.01 | 0.52 |
| 1 / 21 . | 0.00 | 0.00 | 0.04 | 42.90 | 0.00 | 0.00 | 0.11 | 0.51 | 0.03 | 50.45 | -0.01 | 0.00 | 0.00 | -0.03 | 0.71 | -0.10 | -0.01 | 0.61 |
| 1 / 22 . | 0.00 | 0.00 | 0.03 | 42.32 | 0.01 | 0.00 | 0.14 | 0.32 | 0.02 | 49.89 | -0.01 | -0.01 | 0.00 | -0.03 | 1.20 | -0.06 | -0.02 | 0.78 |
| 1 / 23 . | 0.00 | 0.00 | 0.03 | 42.81 | 0.02 | -0.01 | 0.10 | 0.38 | 0.02 | 50.55 | -0.01 | -0.01 | 0.00 | -0.02 | 0.87 | -0.08 | 0.02 | 0.49 |
| 1 / 24 . | 0.00 | 0.00 | 0.05 | 42.84 | 0.01 | 0.01 | 0.14 | 0.30 | 0.01 | 51.12 | 0.00 | 0.00 | 0.00 | -0.02 | 0.83 | -0.04 | 0.01 | 0.57 |
| 1 / 25 . | 0.01 | 0.00 | 0.04 | 43.33 | 0.01 | 0.00 | 0.06 | 0.20 | 0.01 | 50.63 | 0.00 | 0.00 | 0.00 | -0.01 | 0.45 | -0.04 | 0.02 | 0.41 |
| 1 / 26 . | -0.01 | 0.00 | 0.04 | 43.37 | 0.01 | 0.01 | 0.06 | 0.23 | 0.01 | 51.69 | 0.00 | 0.00 | 0.00 | 0.00 | 0.31 | -0.05 | 0.01 | 0.43 |
| 1 / 27 . | 0.00 | 0.00 | 0.04 | 43.40 | -0.01 | 0.00 | 0.08 | 0.30 | 0.01 | 50.70 | 0.02 | 0.00 | 0.00 | 0.00 | 0.54 | -0.06 | 0.00 | 0.39 |
| 1 / 28 . | 0.00 | 0.00 | 0.04 | 43.40 | 0.01 | 0.01 | 0.10 | 0.28 | 0.02 | 50.77 | -0.02 | 0.00 | 0.00 | -0.01 | 0.62 | -0.06 | 0.00 | 0.31 |
| 1 / 29 . | -0.01 | 0.01 | 0.02 | 43.23 | 0.02 | 0.01 | 0.10 | 0.40 | 0.01 | 51.49 | -0.01 | 0.00 | 0.00 | -0.01 | 0.56 | -0.05 | 0.01 | 0.41 |
| 1 / 30 . | 0.00 | 0.00 | 0.06 | 43.54 | 0.00 | 0.00 | 0.11 | 0.63 | 0.01 | 50.63 | 0.00 | -0.01 | 0.01 | 0.00 | 0.43 | -0.04 | 0.02 | 0.29 |
| 1 / 31 . | 0.00 | 0.00 | 0.03 | 43.56 | 0.02 | 0.02 | 0.12 | 0.54 | 0.01 | 51.03 | -0.02 | -0.01 | 0.00 | -0.02 | 0.47 | -0.05 | 0.00 | 0.37 |
| 1 / 32 . | 0.00 | 0.00 | 0.04 | 44.37 | 0.01 | 0.01 | 0.07 | 0.02 | 0.00 | 52.79 | 0.01 | -0.01 | 0.00 | 0.01 | 0.07 | -0.02 | -0.01 | 0.14 |
| 1 / 33 . | -0.01 | 0.00 | 0.04 | 43.46 | -0.02 | 0.02 | 0.12 | 0.47 | 0.00 | 51.33 | 0.01 | 0.00 | 0.01 | -0.01 | 0.23 | -0.04 | -0.02 | 0.60 |
| 1 / 34 . | 0.00 | 0.00 | 0.02 | 44.62 | 0.00 | 0.00 | 0.13 | 0.08 | -0.01 | 52.17 | 0.00 | -0.01 | -0.01 | 0.00 | 0.46 | -0.02 | -0.01 | 0.41 |
| 1 / 35 . | 0.00 | 0.01 | 0.04 | 43.42 | 0.01 | 0.00 | 0.18 | 0.17 | 0.00 | 51.73 | 0.01 | 0.00 | 0.00 | 0.00 | 0.38 | -0.03 | 0.00 | 0.58 |
| 1 / 36 . | 0.00 | 0.00 | 0.00 | 44.47 | -0.01 | 0.01 | 0.02 | 0.29 | 0.00 | 51.92 | 0.01 | 0.01 | -0.01 | 0.01 | 0.06 | -0.03 | 0.00 | 0.43 |
| 1 / 37 . | 0.00 | 0.00 | 0.02 | 44.55 | -0.01 | -0.01 | 0.04 | 0.24 | 0.00 | 52.09 | 0.00 | -0.01 | 0.00 | 0.00 | 0.10 | -0.03 | 0.01 | 0.40 |
| Figure S5 (Area 3, Yr_OR6468) | | | | |  |  |  |  |  |  |  |  |  |  |  |  |  |  |
| Point | P | V | Mn | Fe | Co | Ni | Cu | Zn | Si | S | Cr | Ti | Ca | Mg | As | Sr | Ba | Pb |
| 1 / 1 . | 0.01 | 0.00 | 0.01 | 46.97 | 0.00 | 0.00 | 0.00 | 0.01 | 0.00 | 53.81 | 0.01 | 0.01 | -0.01 | 0.01 | 0.12 | -0.05 | -0.01 | 0.14 |
| 1 / 2 . | 0.00 | 0.00 | 0.02 | 46.76 | -0.01 | 0.00 | 0.00 | 0.01 | 0.04 | 53.58 | 0.02 | -0.01 | -0.01 | 0.00 | 0.12 | -0.17 | -0.01 | 0.14 |
| 1 / 3 . | 0.00 | 0.01 | 0.03 | 46.85 | 0.00 | -0.01 | 0.00 | -0.02 | 0.01 | 53.19 | 0.01 | 0.00 | -0.01 | 0.00 | 0.15 | -0.06 | -0.01 | 0.11 |
| 1 / 4 . | 0.00 | 0.00 | 0.01 | 47.12 | 0.01 | -0.01 | 0.00 | 0.02 | 0.00 | 53.50 | 0.00 | 0.00 | 0.00 | 0.00 | 0.15 | -0.04 | -0.01 | 0.14 |
| 1 / 5 . | 0.01 | 0.00 | 0.05 | 46.32 | 0.01 | 0.00 | -0.01 | 0.02 | 0.01 | 52.95 | 0.00 | 0.00 | -0.01 | 0.00 | 0.15 | -0.05 | 0.01 | 0.30 |
| 1 / 6 . | 0.01 | 0.00 | 0.02 | 46.98 | -0.01 | 0.00 | 0.01 | 0.06 | 0.02 | 52.99 | 0.00 | -0.01 | 0.00 | -0.01 | 0.25 | -0.07 | 0.01 | 0.30 |
| 1 / 7 . | 0.00 | 0.00 | 0.02 | 46.34 | -0.01 | -0.01 | 0.03 | 0.02 | 0.04 | 53.18 | 0.02 | 0.00 | 0.00 | -0.01 | 0.18 | -0.18 | 0.00 | 0.20 |
| 1 / 8 . | 0.00 | 0.01 | 0.02 | 47.22 | -0.01 | 0.01 | 0.02 | 0.00 | 0.01 | 53.43 | -0.02 | 0.00 | 0.00 | 0.00 | 0.07 | -0.03 | 0.01 | 0.14 |
| 1 / 9 . | 0.01 | 0.01 | 0.01 | 47.20 | -0.01 | 0.01 | -0.01 | -0.02 | 0.00 | 53.64 | 0.01 | 0.00 | -0.01 | 0.00 | 0.17 | -0.03 | -0.02 | 0.15 |
| 1 / 10 . | 0.00 | 0.00 | 0.02 | 47.06 | 0.01 | 0.00 | 0.00 | 0.02 | 0.00 | 53.64 | 0.00 | 0.00 | 0.00 | 0.01 | 0.14 | -0.03 | 0.00 | 0.14 |
| 1 / 11 . | 0.00 | 0.00 | 0.00 | 47.02 | 0.01 | -0.01 | 0.01 | -0.02 | 0.00 | 53.66 | 0.00 | 0.00 | -0.02 | 0.00 | 0.23 | -0.04 | 0.02 | 0.15 |
| 1 / 12 . | 0.00 | 0.00 | 0.01 | 47.22 | 0.00 | 0.00 | -0.02 | 0.03 | 0.00 | 53.55 | 0.00 | 0.00 | -0.01 | 0.00 | 0.15 | -0.05 | -0.01 | 0.12 |
| 1 / 13 . | 0.00 | 0.00 | 0.00 | 47.13 | -0.02 | 0.01 | 0.00 | -0.02 | 0.02 | 53.33 | 0.01 | 0.00 | 0.00 | 0.00 | 0.14 | -0.10 | -0.03 | 0.13 |
| 1 / 14 . | 0.00 | -0.01 | 0.01 | 47.28 | -0.01 | 0.00 | -0.02 | -0.01 | 0.00 | 53.45 | -0.01 | -0.01 | -0.01 | 0.00 | 0.19 | -0.03 | -0.02 | 0.13 |
| 1 / 15 . | 0.01 | 0.00 | 0.00 | 46.75 | 0.02 | 0.01 | 0.02 | -0.02 | 0.01 | 52.20 | 0.02 | 0.00 | -0.01 | 0.00 | 0.23 | -0.08 | 0.00 | 0.24 |
| 1 / 16 . | 0.00 | 0.00 | 0.02 | 47.07 | -0.01 | -0.01 | 0.03 | 0.00 | 0.00 | 53.39 | 0.02 | 0.00 | -0.01 | 0.00 | 0.13 | -0.04 | -0.01 | 0.10 |
| 1 / 17 . | 0.00 | 0.01 | 0.02 | 47.09 | -0.01 | 0.00 | 0.01 | 0.00 | 0.01 | 53.47 | 0.00 | 0.00 | 0.00 | 0.00 | 0.13 | -0.05 | 0.00 | 0.13 |
| 1 / 18 . | 0.00 | 0.00 | 0.01 | 47.13 | 0.01 | -0.02 | 0.00 | -0.01 | 0.01 | 53.45 | 0.01 | 0.00 | -0.01 | 0.00 | 0.16 | -0.04 | 0.01 | 0.13 |
| 1 / 19 . | 0.00 | 0.01 | 0.07 | 43.41 | 0.01 | 0.00 | 0.06 | -0.01 | 1.36 | 49.14 | 0.00 | -0.01 | -0.01 | -0.01 | 0.30 | 0.00 | 0.00 | 0.33 |
| 1 / 20 . | 0.00 | 0.01 | 0.02 | 46.97 | 0.00 | 0.00 | -0.01 | 0.00 | 0.00 | 53.26 | 0.00 | 0.00 | 0.00 | 0.00 | 0.26 | -0.04 | 0.02 | 0.22 |
| 1 / 21 . | 0.00 | -0.01 | 0.01 | 46.05 | 0.02 | -0.01 | -0.01 | 0.05 | 0.01 | 52.25 | 0.00 | 0.00 | 0.00 | 0.00 | 0.24 | -0.04 | 0.00 | 1.04 |
| 1 / 22 . | 0.00 | 0.00 | 0.01 | 47.27 | -0.01 | 0.02 | 0.01 | 0.01 | 0.00 | 53.71 | 0.00 | 0.00 | 0.00 | 0.01 | 0.20 | -0.02 | 0.01 | 0.14 |
| 1 / 23 . | 0.01 | 0.00 | 0.01 | 46.97 | 0.02 | 0.00 | -0.01 | 0.02 | 0.01 | 53.30 | 0.00 | 0.00 | 0.00 | -0.01 | 0.32 | -0.03 | 0.01 | 0.13 |
| 1 / 24 . | 0.00 | 0.01 | 0.01 | 46.86 | -0.03 | 0.00 | 0.00 | -0.02 | 0.03 | 53.81 | 0.01 | 0.00 | 0.00 | 0.00 | 0.21 | -0.15 | 0.00 | 0.21 |
| 1 / 25 . | 0.00 | 0.01 | 0.02 | 47.13 | 0.00 | 0.00 | -0.01 | 0.02 | 0.01 | 53.26 | -0.02 | 0.00 | -0.01 | 0.01 | 0.18 | -0.04 | -0.01 | 0.13 |
| 1 / 26 . | 0.01 | 0.00 | 0.02 | 47.16 | -0.01 | 0.00 | -0.01 | -0.04 | 0.01 | 53.54 | 0.00 | -0.01 | -0.01 | 0.00 | 0.19 | -0.04 | -0.01 | 0.09 |
| 1 / 27 . | 0.00 | 0.00 | 0.01 | 47.33 | 0.00 | -0.01 | 0.01 | 0.01 | 0.00 | 53.39 | 0.01 | 0.00 | -0.01 | 0.00 | 0.23 | -0.03 | -0.01 | 0.16 |
| 1 / 28 . | 0.00 | 0.00 | 0.03 | 47.07 | -0.01 | 0.00 | 0.00 | -0.01 | 0.01 | 53.75 | -0.01 | 0.00 | 0.00 | 0.00 | 0.19 | -0.04 | 0.00 | 0.15 |
| Figure S6 (Area 4, Yr_OR6468) | | | | |  |  |  |  |  |  |  |  |  |  |  |  |  |  |
| Point | P | V | Mn | Fe | Co | Ni | Cu | Zn | Si | S | Cr | Ti | Ca | Mg | As | Sr | Ba | Pb |
| 1 / 1 . | 0.00 | 0.00 | 0.02 | 46.76 | -0.01 | 0.00 | 0.00 | 0.01 | 0.03 | 53.92 | 0.00 | -0.01 | 0.00 | 0.00 | 0.24 | -0.07 | 0.02 | 0.21 |
| 1 / 2 . | 0.00 | -0.01 | 0.01 | 46.57 | 0.00 | 0.00 | 0.01 | 0.02 | 0.03 | 53.63 | 0.01 | 0.00 | 0.00 | 0.00 | 0.31 | -0.13 | -0.01 | 0.28 |
| 1 / 3 . | 0.03 | 0.00 | 0.01 | 46.57 | 0.00 | 0.02 | 0.01 | 0.05 | 0.15 | 48.91 | 0.00 | 0.00 | 0.01 | -0.01 | 0.23 | -0.26 | 0.01 | 0.23 |
| 1 / 4 . | 0.00 | 0.00 | 0.00 | 46.68 | 0.01 | -0.01 | -0.01 | 0.05 | 0.01 | 53.25 | 0.01 | 0.00 | -0.01 | -0.01 | 0.27 | -0.04 | -0.02 | 0.19 |
| 1 / 5 . | 0.00 | 0.01 | 0.01 | 47.27 | 0.01 | -0.01 | -0.01 | 0.00 | 0.01 | 53.49 | 0.00 | 0.00 | 0.00 | 0.00 | 0.17 | -0.04 | 0.02 | 0.24 |
| 1 / 6 . | 0.01 | 0.00 | 0.01 | 47.03 | 0.00 | 0.00 | 0.00 | 0.02 | 0.01 | 53.58 | 0.00 | 0.00 | 0.00 | 0.00 | 0.16 | -0.05 | -0.01 | 0.17 |
| 1 / 7 . | 0.00 | 0.00 | 0.01 | 46.63 | -0.01 | 0.01 | 0.02 | 0.01 | 0.07 | 53.42 | 0.00 | -0.01 | 0.00 | 0.00 | 0.20 | -0.17 | -0.04 | 0.21 |
| 1 / 8 . | 0.00 | 0.00 | 0.01 | 46.79 | 0.00 | 0.00 | 0.02 | 0.00 | 0.11 | 53.71 | 0.00 | 0.01 | -0.01 | 0.14 | 0.26 | -0.39 | -0.02 | 0.20 |
| 1 / 9 . | 0.00 | 0.00 | 0.01 | 46.80 | -0.01 | 0.01 | 0.02 | 0.04 | 0.02 | 53.52 | 0.01 | 0.00 | -0.01 | -0.01 | 0.21 | -0.12 | -0.01 | 0.18 |
| 1 / 10 . | 0.00 | -0.01 | 0.02 | 47.16 | -0.01 | -0.01 | 0.01 | 0.01 | 0.00 | 53.88 | 0.00 | 0.01 | -0.01 | 0.00 | 0.11 | -0.06 | 0.01 | 0.14 |
| 1 / 11 . | 0.00 | 0.01 | 0.01 | 47.35 | 0.01 | 0.00 | -0.02 | -0.03 | 0.00 | 53.97 | 0.00 | 0.00 | 0.00 | 0.00 | 0.04 | -0.04 | -0.02 | 0.14 |
| 1 / 12 . | 0.00 | 0.00 | 0.00 | 46.76 | 0.01 | 0.00 | 0.00 | 0.03 | 0.01 | 53.48 | 0.00 | 0.00 | 0.00 | 0.00 | 0.34 | -0.06 | -0.03 | 0.22 |
| 1 / 13 . | 0.00 | 0.00 | 0.01 | 47.07 | 0.01 | 0.00 | 0.00 | 0.04 | 0.01 | 53.66 | -0.01 | -0.01 | 0.00 | 0.00 | 0.22 | -0.03 | 0.00 | 0.18 |
| 1 / 14 . | 0.00 | 0.00 | -0.01 | 47.13 | -0.02 | 0.00 | -0.01 | 0.02 | 0.00 | 53.65 | -0.01 | 0.00 | -0.01 | 0.00 | 0.24 | -0.03 | 0.00 | 0.24 |
| 1 / 15 . | 0.01 | 0.00 | 0.02 | 46.70 | 0.01 | 0.01 | -0.02 | 0.03 | 0.00 | 53.75 | 0.00 | 0.00 | -0.01 | 0.00 | 0.28 | -0.05 | -0.01 | 0.22 |
| 1 / 16 . | 0.00 | 0.00 | 0.02 | 46.95 | -0.01 | -0.02 | 0.00 | 0.04 | 0.01 | 53.42 | -0.01 | 0.00 | 0.00 | 0.00 | 0.16 | -0.06 | 0.01 | 0.15 |
| 1 / 17 . | 0.00 | 0.00 | 0.00 | 47.08 | 0.00 | 0.00 | -0.01 | 0.03 | 0.01 | 53.70 | -0.01 | 0.00 | 0.00 | 0.00 | 0.23 | -0.04 | 0.01 | 0.15 |
| 1 / 18 . | 0.00 | 0.00 | 0.01 | 47.20 | 0.01 | -0.01 | 0.00 | 0.02 | 0.00 | 53.29 | 0.00 | 0.00 | 0.00 | 0.00 | 0.22 | -0.03 | 0.01 | 0.11 |
| 1 / 19 . | 0.00 | 0.00 | 0.02 | 46.61 | 0.00 | 0.00 | 0.02 | 0.01 | 0.50 | 53.07 | 0.00 | 0.00 | -0.01 | 0.00 | 0.27 | 0.00 | 0.02 | 0.25 |
| 1 / 20 . | 0.00 | 0.00 | 0.00 | 46.74 | 0.01 | 0.00 | 0.00 | 0.06 | 0.04 | 53.34 | -0.01 | 0.00 | 0.00 | 0.00 | 0.19 | -0.14 | 0.01 | 0.24 |
| 1 / 21 . | -0.01 | 0.00 | 0.01 | 47.14 | 0.00 | 0.00 | -0.01 | 0.02 | 0.00 | 53.72 | -0.01 | -0.01 | 0.00 | 0.01 | 0.20 | -0.03 | 0.02 | 0.18 |
| 1 / 22 . | 0.00 | -0.01 | 0.00 | 46.99 | 0.01 | 0.01 | 0.01 | 0.00 | 0.01 | 53.34 | -0.02 | -0.01 | 0.00 | 0.00 | 0.22 | -0.04 | -0.01 | 0.18 |
| 1 / 23 . | 0.00 | 0.00 | 0.01 | 46.57 | 0.00 | 0.00 | 0.01 | 0.03 | 0.07 | 53.56 | -0.01 | 0.00 | 0.00 | 0.00 | 0.25 | -0.24 | 0.01 | 0.21 |
| 1 / 24 . | 0.00 | 0.01 | -0.01 | 46.77 | 0.00 | 0.00 | 0.01 | 0.02 | 0.02 | 53.20 | 0.01 | 0.01 | -0.01 | 0.00 | 0.22 | -0.06 | 0.00 | 0.27 |
| 1 / 25 . | 0.00 | -0.01 | 0.02 | 46.96 | -0.01 | -0.02 | 0.00 | 0.04 | 0.01 | 53.59 | 0.01 | 0.00 | -0.01 | 0.01 | 0.24 | -0.04 | 0.01 | 0.19 |
| 1 / 26 . | 0.00 | 0.00 | 0.01 | 47.03 | 0.00 | 0.00 | 0.03 | 0.02 | 0.02 | 53.47 | 0.01 | 0.00 | 0.00 | -0.01 | 0.16 | -0.06 | 0.00 | 0.18 |
| 1 / 27 . | 0.01 | 0.00 | 0.00 | 46.79 | 0.01 | 0.02 | 0.01 | 0.02 | 0.15 | 52.47 | 0.00 | 0.00 | 0.00 | -0.01 | 0.31 | -0.36 | -0.02 | 0.25 |
| 1 / 28 . | 0.00 | 0.00 | 0.02 | 43.60 | 0.02 | 0.00 | 0.03 | 0.03 | 0.14 | 49.97 | -0.01 | -0.01 | 0.00 | 0.00 | 0.35 | -0.45 | 0.01 | 0.30 |
| 1 / 29 . | 0.00 | 0.00 | 0.02 | 46.98 | -0.04 | -0.01 | 0.01 | 0.00 | 0.00 | 53.58 | 0.00 | -0.01 | -0.01 | 0.00 | 0.17 | -0.01 | 0.01 | 0.15 |
| 1 / 30 . | -0.01 | 0.00 | 0.01 | 46.94 | -0.01 | 0.00 | -0.01 | 0.04 | 0.00 | 53.52 | 0.00 | 0.00 | -0.02 | 0.00 | 0.22 | -0.05 | 0.00 | 0.18 |
| 1 / 31 . | 0.01 | 0.00 | 0.04 | 46.94 | 0.01 | 0.01 | -0.01 | 0.04 | 0.01 | 53.46 | -0.02 | 0.00 | 0.00 | 0.00 | 0.22 | -0.04 | -0.01 | 0.20 |
| 1 / 32 . | 0.00 | -0.01 | 0.01 | 46.89 | 0.00 | 0.01 | -0.01 | 0.01 | 0.01 | 53.39 | 0.01 | 0.00 | 0.00 | 0.00 | 0.22 | -0.03 | -0.01 | 0.20 |
| 1 / 33 . | 0.00 | 0.01 | 0.02 | 46.79 | 0.00 | -0.01 | 0.01 | 0.01 | 0.02 | 53.42 | -0.02 | -0.01 | 0.00 | -0.01 | 0.23 | -0.08 | 0.02 | 0.18 |
| 1 / 34 . | 0.00 | 0.00 | 0.03 | 46.83 | 0.00 | -0.01 | -0.01 | 0.05 | 0.00 | 53.43 | 0.01 | 0.00 | -0.01 | 0.00 | 0.22 | -0.03 | 0.00 | 0.21 |
| 1 / 35 . | 0.00 | 0.00 | 0.00 | 47.06 | 0.01 | 0.00 | 0.00 | 0.02 | 0.00 | 53.64 | 0.00 | 0.01 | -0.01 | -0.01 | 0.19 | -0.03 | 0.00 | 0.15 |
| 1 / 36 . | 0.01 | 0.00 | 0.02 | 47.03 | 0.00 | -0.01 | 0.00 | 0.01 | 0.00 | 53.85 | -0.01 | -0.01 | -0.01 | 0.01 | 0.07 | -0.05 | 0.01 | 0.14 |
| 1 / 37 . | 0.00 | 0.00 | 0.04 | 47.02 | 0.01 | 0.00 | 0.01 | -0.02 | 0.04 | 53.89 | -0.01 | 0.00 | -0.01 | 0.00 | 0.08 | -0.12 | -0.01 | 0.12 |
| 1 / 38 . | 0.00 | 0.00 | 0.02 | 47.22 | -0.02 | 0.01 | 0.00 | 0.01 | 0.00 | 53.41 | 0.00 | 0.00 | -0.01 | 0.00 | 0.19 | -0.03 | 0.01 | 0.16 |
| 1 / 39 . | 0.00 | 0.00 | 0.00 | 46.98 | -0.02 | 0.00 | -0.01 | 0.02 | 0.00 | 53.22 | -0.01 | 0.00 | 0.00 | 0.00 | 0.22 | -0.04 | 0.01 | 0.12 |
| 1 / 40 . | -0.01 | 0.00 | 0.03 | 46.91 | 0.00 | -0.01 | -0.01 | 0.04 | 0.00 | 53.40 | 0.01 | 0.00 | -0.01 | 0.00 | 0.21 | -0.03 | -0.02 | 0.12 |

**Table S5** Detection limits (in weight %) for EPMA analyses of pyrite composition around Yaman Kasy microfossils and in adjacent pyrite that does not contain them (grey rows).

| Detection limits - Figure S3 (Area 1, Yr_61633) | | | | | | |  |  |  |  |  |  |  |  |  |  |  |  |
| --- | --- | --- | --- | --- | --- | --- | --- | --- | --- | --- | --- | --- | --- | --- | --- | --- | --- | --- |
| Point | P | V | Mn | Fe | Co | Ni | Cu | Zn | Si | S | Cr | Ti | Ca | Mg | As | Sr | Ba | Pb |
| 1 / 1 . | 0.02 | 0.02 | 0.03 | 0.04 | -0.01 | 0.03 | 0.04 | 0.06 | 0.01 | 0.05 | 0.04 | 0.02 | 0.02 | 0.01 | 0.02 | 0.03 | 0.05 | 0.05 |
| 1 / 2 . | 0.01 | 0.02 | 0.03 | 0.04 | -0.02 | 0.03 | 0.04 | 0.06 | 0.01 | 0.05 | 0.04 | 0.02 | 0.02 | 0.01 | 0.02 | 0.03 | 0.05 | 0.05 |
| 1 / 3 . | 0.01 | 0.02 | 0.03 | 0.04 | 0.00 | 0.03 | 0.04 | 0.06 | 0.01 | 0.05 | 0.04 | 0.02 | 0.02 | 0.01 | 0.02 | 0.03 | 0.05 | 0.05 |
| 1 / 4 . | 0.01 | 0.02 | 0.03 | 0.04 | 0.01 | 0.03 | 0.04 | 0.05 | 0.01 | 0.05 | 0.04 | 0.02 | 0.02 | 0.01 | 0.02 | 0.03 | 0.05 | 0.05 |
| 1 / 5 . | 0.01 | 0.02 | 0.03 | 0.04 | -0.03 | 0.03 | 0.04 | 0.06 | 0.01 | 0.06 | 0.04 | 0.02 | 0.02 | 0.01 | 0.02 | 0.03 | 0.05 | 0.05 |
| 1 / 6 . | 0.01 | 0.02 | 0.03 | 0.04 | -0.02 | 0.03 | 0.04 | 0.06 | 0.01 | 0.05 | 0.03 | 0.02 | 0.01 | 0.01 | 0.02 | 0.03 | 0.05 | 0.05 |
| 1 / 7 . | 0.01 | 0.02 | 0.03 | 0.04 | 0.01 | 0.03 | 0.04 | 0.05 | 0.01 | 0.05 | 0.04 | 0.02 | 0.01 | 0.01 | 0.02 | 0.03 | 0.05 | 0.05 |
| 1 / 8 . | 0.02 | 0.02 | 0.03 | 0.04 | 0.01 | 0.03 | 0.04 | 0.06 | 0.01 | 0.05 | 0.03 | 0.02 | 0.02 | 0.01 | 0.02 | 0.03 | 0.05 | 0.05 |
| 1 / 9 . | 0.01 | 0.02 | 0.03 | 0.04 | 0.00 | 0.03 | 0.04 | 0.06 | 0.01 | 0.05 | 0.04 | 0.02 | 0.02 | 0.01 | 0.02 | 0.03 | 0.05 | 0.05 |
| 1 / 10 . | 0.01 | 0.02 | 0.03 | 0.04 | 0.00 | 0.03 | 0.04 | 0.05 | 0.01 | 0.06 | 0.04 | 0.02 | 0.02 | 0.01 | 0.02 | 0.03 | 0.05 | 0.05 |
| 1 / 11 . | 0.01 | 0.02 | 0.03 | 0.04 | -0.01 | 0.03 | 0.04 | 0.06 | 0.01 | 0.05 | 0.04 | 0.02 | 0.02 | 0.01 | 0.02 | 0.03 | 0.05 | 0.05 |
| 1 / 12 . | 0.01 | 0.02 | 0.03 | 0.04 | 0.01 | 0.03 | 0.04 | 0.06 | 0.01 | 0.06 | 0.03 | 0.02 | 0.02 | 0.01 | 0.02 | 0.03 | 0.05 | 0.05 |
| 1 / 13 . | 0.01 | 0.02 | 0.03 | 0.04 | -0.02 | 0.03 | 0.04 | 0.06 | 0.01 | 0.05 | 0.04 | 0.02 | 0.02 | 0.01 | 0.02 | 0.03 | 0.05 | 0.05 |
| 1 / 14 . | 0.01 | 0.02 | 0.03 | 0.04 | 0.01 | 0.03 | 0.04 | 0.05 | 0.01 | 0.05 | 0.04 | 0.02 | 0.02 | 0.01 | 0.02 | 0.03 | 0.05 | 0.05 |
| 1 / 15 . | 0.01 | 0.02 | 0.03 | 0.04 | 0.01 | 0.03 | 0.04 | 0.05 | 0.01 | 0.05 | 0.04 | 0.02 | 0.02 | 0.01 | 0.02 | 0.03 | 0.05 | 0.05 |
| 1 / 16 . | 0.02 | 0.02 | 0.03 | 0.04 | 0.00 | 0.03 | 0.04 | 0.05 | 0.01 | 0.06 | 0.04 | 0.02 | 0.02 | 0.01 | 0.02 | 0.03 | 0.05 | 0.05 |
| 1 / 17 . | 0.01 | 0.02 | 0.03 | 0.04 | -0.01 | 0.03 | 0.04 | 0.05 | 0.01 | 0.06 | 0.04 | 0.02 | 0.02 | 0.01 | 0.02 | 0.03 | 0.05 | 0.05 |
| 1 / 18 . | 0.01 | 0.02 | 0.03 | 0.04 | 0.00 | 0.03 | 0.04 | 0.05 | 0.01 | 0.05 | 0.04 | 0.02 | 0.02 | 0.01 | 0.02 | 0.03 | 0.05 | 0.05 |
| 1 / 19 . | 0.02 | 0.02 | 0.03 | 0.04 | -0.02 | 0.03 | 0.04 | 0.06 | 0.01 | 0.05 | 0.04 | 0.02 | 0.02 | 0.01 | 0.02 | 0.03 | 0.05 | 0.05 |
| 1 / 20 . | 0.02 | 0.02 | 0.03 | 0.04 | 0.00 | 0.03 | 0.04 | 0.05 | 0.01 | 0.05 | 0.04 | 0.02 | 0.02 | 0.01 | 0.02 | 0.03 | 0.05 | 0.05 |
| 1 / 21 . | 0.01 | 0.02 | 0.03 | 0.04 | 0.00 | 0.03 | 0.04 | 0.06 | 0.01 | 0.05 | 0.03 | 0.02 | 0.01 | 0.01 | 0.02 | 0.03 | 0.05 | 0.05 |
| 1 / 22 . | 0.01 | 0.02 | 0.03 | 0.04 | -0.01 | 0.03 | 0.04 | 0.06 | 0.01 | 0.06 | 0.04 | 0.02 | 0.02 | 0.01 | 0.02 | 0.03 | 0.05 | 0.05 |
| 1 / 23 . | 0.01 | 0.02 | 0.03 | 0.04 | 0.00 | 0.03 | 0.04 | 0.06 | 0.01 | 0.05 | 0.04 | 0.02 | 0.02 | 0.01 | 0.02 | 0.03 | 0.05 | 0.05 |
| 1 / 24 . | 0.01 | 0.02 | 0.03 | 0.04 | 0.00 | 0.03 | 0.04 | 0.05 | 0.01 | 0.06 | 0.03 | 0.02 | 0.02 | 0.01 | 0.02 | 0.03 | 0.05 | 0.05 |
| 1 / 25 . | 0.01 | 0.01 | 0.03 | 0.04 | 0.00 | 0.03 | 0.04 | 0.05 | 0.01 | 0.05 | 0.04 | 0.02 | 0.02 | 0.01 | 0.02 | 0.03 | 0.05 | 0.05 |
| 1 / 26 . | 0.02 | 0.02 | 0.03 | 0.04 | 0.00 | 0.03 | 0.04 | 0.06 | 0.01 | 0.05 | 0.04 | 0.02 | 0.02 | 0.01 | 0.02 | 0.03 | 0.05 | 0.05 |
| 1 / 27 . | 0.02 | 0.02 | 0.03 | 0.04 | 0.01 | 0.03 | 0.04 | 0.06 | 0.01 | 0.06 | 0.03 | 0.02 | 0.02 | 0.01 | 0.02 | 0.04 | 0.05 | 0.05 |
| 1 / 28 . | 0.01 | 0.02 | 0.03 | 0.04 | 0.00 | 0.03 | 0.04 | 0.05 | 0.01 | 0.06 | 0.04 | 0.02 | 0.02 | 0.01 | 0.02 | 0.03 | 0.05 | 0.05 |
| 1 / 29 . | 0.01 | 0.02 | 0.03 | 0.04 | 0.01 | 0.03 | 0.04 | 0.06 | 0.01 | 0.05 | 0.03 | 0.02 | 0.02 | 0.01 | 0.03 | 0.03 | 0.05 | 0.05 |
| 1 / 30 . | 0.01 | 0.02 | 0.03 | 0.04 | 0.00 | 0.03 | 0.04 | 0.06 | 0.01 | 0.05 | 0.04 | 0.02 | 0.02 | 0.01 | 0.03 | 0.03 | 0.05 | 0.05 |
| Detection limits - Figure S4 (Area 2, Yr_61633) | | | | | | |  |  |  |  |  |  |  |  |  |  |  |  |
| Point | P | V | Mn | Fe | Co | Ni | Cu | Zn | Si | S | Cr | Ti | Ca | Mg | As | Sr | Ba | Pb |
| 1 / 1 . | 0.02 | 0.02 | 0.03 | 0.04 | 0.01 | 0.03 | 0.04 | 0.05 | 0.01 | 0.06 | 0.04 | 0.02 | 0.02 | 0.01 | 0.02 | 0.03 | 0.05 | 0.05 |
| 1 / 2 . | 0.02 | 0.02 | 0.03 | 0.04 | 0.00 | 0.03 | 0.04 | 0.05 | 0.01 | 0.05 | 0.04 | 0.02 | 0.02 | 0.01 | 0.02 | 0.03 | 0.05 | 0.05 |
| 1 / 3 . | 0.01 | 0.02 | 0.03 | 0.04 | 0.00 | 0.03 | 0.04 | 0.05 | 0.01 | 0.05 | 0.04 | 0.02 | 0.01 | 0.01 | 0.02 | 0.03 | 0.05 | 0.05 |
| 1 / 4 . | 0.02 | 0.02 | 0.03 | 0.04 | 0.01 | 0.03 | 0.04 | 0.05 | 0.01 | 0.05 | 0.04 | 0.02 | 0.02 | 0.01 | 0.02 | 0.03 | 0.05 | 0.05 |
| 1 / 5 . | 0.01 | 0.02 | 0.03 | 0.04 | 0.00 | 0.03 | 0.04 | 0.05 | 0.01 | 0.05 | 0.04 | 0.02 | 0.02 | 0.01 | 0.02 | 0.03 | 0.05 | 0.05 |
| 1 / 6 . | 0.01 | 0.02 | 0.03 | 0.04 | -0.02 | 0.03 | 0.04 | 0.05 | 0.01 | 0.05 | 0.04 | 0.02 | 0.02 | 0.01 | 0.03 | 0.03 | 0.05 | 0.05 |
| 1 / 7 . | 0.01 | 0.02 | 0.03 | 0.04 | -0.01 | 0.03 | 0.04 | 0.05 | 0.01 | 0.05 | 0.04 | 0.02 | 0.02 | 0.01 | 0.02 | 0.03 | 0.05 | 0.05 |
| 1 / 8 . | 0.01 | 0.02 | 0.03 | 0.04 | 0.01 | 0.03 | 0.04 | 0.05 | 0.01 | 0.06 | 0.04 | 0.02 | 0.02 | 0.01 | 0.02 | 0.03 | 0.05 | 0.05 |
| 1 / 9 . | 0.02 | 0.02 | 0.03 | 0.04 | 0.00 | 0.03 | 0.04 | 0.05 | 0.01 | 0.05 | 0.04 | 0.02 | 0.01 | 0.01 | 0.02 | 0.03 | 0.05 | 0.05 |
| 1 / 10 . | 0.02 | 0.02 | 0.03 | 0.04 | 0.01 | 0.03 | 0.04 | 0.06 | 0.01 | 0.05 | 0.04 | 0.02 | 0.02 | 0.01 | 0.02 | 0.03 | 0.05 | 0.05 |
| 1 / 11 . | 0.01 | 0.02 | 0.03 | 0.04 | 0.01 | 0.03 | 0.04 | 0.06 | 0.01 | 0.05 | 0.03 | 0.02 | 0.02 | 0.01 | 0.02 | 0.03 | 0.05 | 0.05 |
| 1 / 12 . | 0.02 | 0.02 | 0.03 | 0.04 | 0.01 | 0.03 | 0.04 | 0.06 | 0.01 | 0.06 | 0.04 | 0.02 | 0.02 | 0.01 | 0.02 | 0.03 | 0.05 | 0.05 |
| 1 / 13 . | 0.01 | 0.02 | 0.03 | 0.04 | 0.00 | 0.03 | 0.04 | 0.06 | 0.01 | 0.06 | 0.04 | 0.02 | 0.02 | 0.01 | 0.02 | 0.03 | 0.05 | 0.05 |
| 1 / 14 . | 0.02 | 0.02 | 0.03 | 0.04 | 0.01 | 0.03 | 0.04 | 0.06 | 0.01 | 0.06 | 0.04 | 0.02 | 0.02 | 0.01 | 0.02 | 0.03 | 0.05 | 0.06 |
| 1 / 15 . | 0.02 | 0.02 | 0.03 | 0.04 | -0.01 | 0.03 | 0.04 | 0.06 | 0.01 | 0.05 | 0.04 | 0.02 | 0.01 | 0.01 | 0.02 | 0.03 | 0.05 | 0.05 |
| 1 / 16 . | 0.01 | 0.02 | 0.03 | 0.04 | 0.00 | 0.03 | 0.04 | 0.05 | 0.01 | 0.05 | 0.03 | 0.02 | 0.02 | 0.01 | 0.02 | 0.03 | 0.05 | 0.05 |
| 1 / 17 . | 0.01 | 0.02 | 0.03 | 0.04 | 0.00 | 0.03 | 0.04 | 0.05 | 0.01 | 0.06 | 0.04 | 0.02 | 0.02 | 0.01 | 0.02 | 0.03 | 0.05 | 0.05 |
| 1 / 18 . | 0.02 | 0.02 | 0.03 | 0.04 | 0.00 | 0.03 | 0.04 | 0.05 | 0.01 | 0.06 | 0.04 | 0.02 | 0.02 | 0.01 | 0.02 | 0.03 | 0.05 | 0.05 |
| 1 / 19 . | 0.02 | 0.02 | 0.03 | 0.04 | 0.00 | 0.03 | 0.04 | 0.06 | 0.01 | 0.05 | 0.04 | 0.02 | 0.02 | 0.01 | 0.02 | 0.03 | 0.05 | 0.05 |
| 1 / 20 . | 0.02 | 0.01 | 0.03 | 0.04 | 0.00 | 0.03 | 0.04 | 0.06 | 0.01 | 0.05 | 0.04 | 0.02 | 0.01 | 0.01 | 0.02 | 0.03 | 0.05 | 0.06 |
| 1 / 21 . | 0.02 | 0.02 | 0.03 | 0.04 | 0.00 | 0.03 | 0.04 | 0.05 | 0.01 | 0.05 | 0.04 | 0.02 | 0.02 | 0.01 | 0.02 | 0.03 | 0.05 | 0.05 |
| 1 / 22 . | 0.02 | 0.02 | 0.03 | 0.04 | 0.00 | 0.03 | 0.04 | 0.06 | 0.01 | 0.05 | 0.04 | 0.02 | 0.02 | 0.01 | 0.02 | 0.03 | 0.05 | 0.05 |
| 1 / 23 . | 0.02 | 0.02 | 0.03 | 0.04 | 0.01 | 0.03 | 0.04 | 0.05 | 0.01 | 0.05 | 0.04 | 0.02 | 0.02 | 0.01 | 0.02 | 0.03 | 0.05 | 0.05 |
| 1 / 24 . | 0.01 | 0.02 | 0.03 | 0.04 | 0.01 | 0.03 | 0.04 | 0.05 | 0.01 | 0.05 | 0.04 | 0.02 | 0.02 | 0.01 | 0.03 | 0.03 | 0.05 | 0.05 |
| 1 / 25 . | 0.01 | 0.02 | 0.03 | 0.04 | 0.01 | 0.03 | 0.04 | 0.05 | 0.01 | 0.05 | 0.04 | 0.02 | 0.02 | 0.01 | 0.02 | 0.03 | 0.05 | 0.05 |
| 1 / 26 . | 0.02 | 0.02 | 0.03 | 0.04 | 0.00 | 0.03 | 0.04 | 0.05 | 0.01 | 0.05 | 0.04 | 0.02 | 0.02 | 0.01 | 0.02 | 0.03 | 0.05 | 0.06 |
| 1 / 27 . | 0.01 | 0.02 | 0.03 | 0.04 | -0.01 | 0.03 | 0.04 | 0.05 | 0.01 | 0.05 | 0.03 | 0.02 | 0.02 | 0.01 | 0.02 | 0.03 | 0.05 | 0.05 |
| 1 / 28 . | 0.02 | 0.02 | 0.03 | 0.04 | 0.00 | 0.03 | 0.04 | 0.06 | 0.01 | 0.06 | 0.04 | 0.02 | 0.02 | 0.01 | 0.02 | 0.03 | 0.05 | 0.05 |
| 1 / 29 . | 0.02 | 0.02 | 0.03 | 0.04 | 0.01 | 0.03 | 0.04 | 0.06 | 0.01 | 0.05 | 0.04 | 0.02 | 0.02 | 0.01 | 0.02 | 0.03 | 0.05 | 0.05 |
| 1 / 30 . | 0.01 | 0.02 | 0.03 | 0.04 | 0.00 | 0.03 | 0.04 | 0.06 | 0.01 | 0.05 | 0.04 | 0.02 | 0.02 | 0.01 | 0.02 | 0.03 | 0.05 | 0.05 |
| 1 / 31 . | 0.02 | 0.02 | 0.03 | 0.04 | 0.01 | 0.03 | 0.04 | 0.05 | 0.01 | 0.05 | 0.04 | 0.02 | 0.02 | 0.01 | 0.02 | 0.03 | 0.05 | 0.05 |
| 1 / 32 . | 0.01 | 0.02 | 0.03 | 0.04 | 0.00 | 0.03 | 0.04 | 0.05 | 0.01 | 0.06 | 0.04 | 0.02 | 0.02 | 0.01 | 0.02 | 0.03 | 0.05 | 0.05 |
| 1 / 33 . | 0.02 | 0.02 | 0.03 | 0.04 | -0.04 | 0.03 | 0.04 | 0.06 | 0.01 | 0.05 | 0.04 | 0.02 | 0.02 | 0.01 | 0.02 | 0.03 | 0.05 | 0.05 |
| 1 / 34 . | 0.01 | 0.00 | 0.03 | 0.04 | 0.00 | 0.03 | 0.04 | 0.05 | 0.01 | 0.06 | 0.04 | 0.02 | 0.02 | 0.01 | 0.02 | 0.03 | 0.05 | 0.05 |
| 1 / 35 . | 0.01 | 0.02 | 0.03 | 0.04 | 0.01 | 0.03 | 0.04 | 0.05 | 0.01 | 0.05 | 0.04 | 0.02 | 0.02 | 0.01 | 0.02 | 0.03 | 0.05 | 0.05 |
| 1 / 36 . | 0.01 | 0.02 | 0.03 | 0.04 | -0.02 | 0.03 | 0.04 | 0.06 | 0.01 | 0.05 | 0.04 | 0.02 | 0.02 | 0.01 | 0.02 | 0.03 | 0.05 | 0.05 |
| 1 / 37 . | 0.02 | 0.02 | 0.03 | 0.04 | -0.01 | 0.03 | 0.04 | 0.06 | 0.01 | 0.05 | 0.04 | 0.02 | 0.02 | 0.01 | 0.02 | 0.03 | 0.05 | 0.06 |
| Detection limits - Figure S5 (Area 3, Yr_OR6468) | | | | | | | |  |  |  |  |  |  |  |  |  |  |  |
| Point | P | V | Mn | Fe | Co | Ni | Cu | Zn | Si | S | Cr | Ti | Ca | Mg | As | Sr | Ba | Pb |
| 1 / 1 . | 0.01 | 0.02 | 0.03 | 0.04 | 0.00 | 0.03 | 0.04 | 0.05 | 0.01 | 0.06 | 0.04 | 0.02 | 0.02 | 0.01 | 0.02 | 0.03 | 0.05 | 0.05 |
| 1 / 2 . | 0.02 | 0.02 | 0.03 | 0.04 | -0.01 | 0.03 | 0.04 | 0.05 | 0.01 | 0.06 | 0.04 | 0.02 | 0.02 | 0.01 | 0.02 | 0.03 | 0.05 | 0.05 |
| 1 / 3 . | 0.01 | 0.02 | 0.03 | 0.04 | 0.00 | 0.03 | 0.04 | 0.06 | 0.01 | 0.05 | 0.04 | 0.02 | 0.02 | 0.01 | 0.02 | 0.03 | 0.05 | 0.06 |
| 1 / 4 . | 0.01 | 0.02 | 0.03 | 0.04 | 0.00 | 0.03 | 0.09 | 0.06 | 0.01 | 0.05 | 0.04 | 0.02 | 0.02 | 0.01 | 0.02 | 0.03 | 0.05 | 0.06 |
| 1 / 5 . | 0.01 | 0.02 | 0.03 | 0.04 | 0.01 | 0.03 | 0.04 | 0.06 | 0.01 | 0.06 | 0.04 | 0.02 | 0.02 | 0.01 | 0.02 | 0.03 | 0.05 | 0.05 |
| 1 / 6 . | 0.01 | 0.02 | 0.03 | 0.04 | -0.01 | 0.03 | 0.04 | 0.06 | 0.01 | 0.06 | 0.04 | 0.02 | 0.02 | 0.01 | 0.02 | 0.03 | 0.05 | 0.05 |
| 1 / 7 . | 0.01 | 0.02 | 0.03 | 0.04 | -0.01 | 0.03 | 0.04 | 0.05 | 0.01 | 0.06 | 0.03 | 0.02 | 0.02 | 0.01 | 0.03 | 0.03 | 0.05 | 0.05 |
| 1 / 8 . | 0.01 | 0.02 | 0.03 | 0.04 | -0.02 | 0.03 | 0.04 | 0.06 | 0.01 | 0.05 | 0.04 | 0.02 | 0.02 | 0.01 | 0.03 | 0.03 | 0.05 | 0.05 |
| 1 / 9 . | 0.01 | 0.02 | 0.03 | 0.04 | -0.01 | 0.03 | 0.04 | 0.06 | 0.01 | 0.05 | 0.04 | 0.02 | 0.02 | 0.01 | 0.02 | 0.03 | 0.05 | 0.05 |
| 1 / 10 . | 0.01 | 0.02 | 0.03 | 0.04 | 0.01 | 0.03 | 0.04 | 0.06 | 0.01 | 0.05 | 0.04 | 0.02 | 0.02 | 0.01 | 0.03 | 0.03 | 0.05 | 0.05 |
| 1 / 11 . | 0.01 | 0.02 | 0.03 | 0.04 | 0.01 | 0.03 | 0.04 | 0.06 | 0.01 | 0.05 | 0.04 | 0.02 | 0.02 | 0.01 | 0.02 | 0.03 | 0.05 | 0.05 |
| 1 / 12 . | 0.02 | 0.02 | 0.03 | 0.04 | 0.00 | 0.03 | 0.04 | 0.05 | 0.01 | 0.05 | 0.04 | 0.02 | 0.02 | 0.01 | 0.02 | 0.03 | 0.05 | 0.05 |
| 1 / 13 . | 0.01 | 0.02 | 0.03 | 0.04 | -0.02 | 0.03 | 0.04 | 0.06 | 0.01 | 0.06 | 0.04 | 0.02 | 0.02 | 0.01 | 0.02 | 0.03 | 0.05 | 0.05 |
| 1 / 14 . | 0.01 | 0.02 | 0.03 | 0.04 | 0.00 | 0.03 | 0.04 | 0.06 | 0.01 | 0.06 | 0.04 | 0.02 | 0.02 | 0.01 | 0.02 | 0.03 | 0.05 | 0.05 |
| 1 / 15 . | 0.01 | 0.02 | 0.03 | 0.04 | 0.01 | 0.03 | 0.04 | 0.06 | 0.01 | 0.06 | 0.04 | 0.02 | 0.02 | 0.01 | 0.02 | 0.03 | 0.05 | 0.05 |
| 1 / 16 . | 0.01 | 0.02 | 0.03 | 0.04 | -0.01 | 0.03 | 0.04 | 0.05 | 0.01 | 0.05 | 0.04 | 0.02 | 0.02 | 0.01 | 0.02 | 0.03 | 0.05 | 0.05 |
| 1 / 17 . | 0.01 | 0.02 | 0.03 | 0.04 | -0.01 | 0.03 | 0.04 | 0.06 | 0.01 | 0.05 | 0.04 | 0.02 | 0.02 | 0.01 | 0.02 | 0.03 | 0.05 | 0.05 |
| 1 / 18 . | 0.02 | 0.02 | 0.03 | 0.04 | 0.01 | 0.03 | 0.04 | 0.06 | 0.01 | 0.05 | 0.04 | 0.02 | 0.02 | 0.01 | 0.02 | 0.03 | 0.05 | 0.05 |
| 1 / 19 . | 0.01 | 0.02 | 0.03 | 0.04 | 0.01 | 0.03 | 0.04 | 0.06 | 0.01 | 0.05 | 0.03 | 0.02 | 0.02 | 0.01 | 0.02 | 0.00 | 0.05 | 0.05 |
| 1 / 20 . | 0.02 | 0.02 | 0.03 | 0.04 | 0.00 | 0.03 | 0.04 | 0.06 | 0.01 | 0.05 | 0.04 | 0.02 | 0.02 | 0.01 | 0.02 | 0.03 | 0.05 | 0.05 |
| 1 / 21 . | 0.02 | 0.02 | 0.03 | 0.04 | 0.01 | 0.03 | 0.04 | 0.06 | 0.01 | 0.05 | 0.04 | 0.02 | 0.02 | 0.01 | 0.02 | 0.03 | 0.05 | 0.05 |
| 1 / 22 . | 0.01 | 0.02 | 0.03 | 0.04 | -0.01 | 0.03 | 0.04 | 0.06 | 0.01 | 0.06 | 0.04 | 0.02 | 0.02 | 0.01 | 0.02 | 0.03 | 0.05 | 0.05 |
| 1 / 23 . | 0.01 | 0.02 | 0.03 | 0.04 | 0.01 | 0.03 | 0.04 | 0.05 | 0.01 | 0.05 | 0.04 | 0.02 | 0.02 | 0.01 | 0.02 | 0.03 | 0.05 | 0.05 |
| 1 / 24 . | 0.01 | 0.02 | 0.03 | 0.04 | -0.05 | 0.03 | 0.04 | 0.06 | 0.01 | 0.06 | 0.04 | 0.02 | 0.02 | 0.01 | 0.02 | 0.03 | 0.05 | 0.05 |
| 1 / 25 . | 0.01 | 0.02 | 0.03 | 0.04 | 0.00 | 0.03 | 0.04 | 0.05 | 0.01 | 0.05 | 0.04 | 0.02 | 0.02 | 0.01 | 0.02 | 0.03 | 0.05 | 0.05 |
| 1 / 26 . | 0.01 | 0.02 | 0.03 | 0.04 | -0.01 | 0.03 | 0.04 | 0.06 | 0.01 | 0.06 | 0.04 | 0.02 | 0.02 | 0.01 | 0.02 | 0.03 | 0.05 | 0.06 |
| 1 / 27 . | 0.02 | 0.02 | 0.03 | 0.04 | 0.00 | 0.03 | 0.04 | 0.05 | 0.01 | 0.05 | 0.04 | 0.02 | 0.02 | 0.01 | 0.02 | 0.03 | 0.05 | 0.05 |
| 1 / 28 . | 0.02 | 0.01 | 0.03 | 0.04 | -0.02 | 0.03 | 0.04 | 0.06 | 0.01 | 0.06 | 0.04 | 0.02 | 0.02 | 0.01 | 0.02 | 0.03 | 0.05 | 0.05 |
| Detection limits - Figure S6 (Area 4, Yr_OR6468) | | | | | | | |  |  |  |  |  |  |  |  |  |  |  |
| Point | P | V | Mn | Fe | Co | Ni | Cu | Zn | Si | S | Cr | Ti | Ca | Mg | As | Sr | Ba | Pb |
| 1 / 1 . | 0.01 | 0.01 | 0.03 | 0.04 | -0.01 | 0.03 | 0.04 | 0.06 | 0.01 | 0.06 | 0.04 | 0.02 | 0.02 | 0.01 | 0.02 | 0.03 | 0.05 | 0.05 |
| 1 / 2 . | 0.01 | 0.02 | 0.03 | 0.04 | 0.00 | 0.03 | 0.04 | 0.06 | 0.01 | 0.06 | 0.04 | 0.02 | 0.02 | 0.01 | 0.02 | 0.03 | 0.05 | 0.05 |
| 1 / 3 . | 0.02 | 0.02 | 0.03 | 0.04 | 0.00 | 0.03 | 0.04 | 0.05 | 0.01 | 0.05 | 0.04 | 0.02 | 0.02 | 0.01 | 0.02 | 0.04 | 0.05 | 0.05 |
| 1 / 4 . | 0.02 | 0.02 | 0.04 | 0.04 | 0.01 | 0.03 | 0.04 | 0.05 | 0.01 | 0.06 | 0.04 | 0.02 | 0.02 | 0.01 | 0.02 | 0.03 | 0.05 | 0.06 |
| 1 / 5 . | 0.01 | 0.02 | 0.03 | 0.04 | 0.01 | 0.03 | 0.04 | 0.06 | 0.01 | 0.06 | 0.04 | 0.02 | 0.02 | 0.01 | 0.02 | 0.03 | 0.05 | 0.05 |
| 1 / 6 . | 0.01 | 0.02 | 0.03 | 0.04 | 0.00 | 0.03 | 0.04 | 0.06 | 0.01 | 0.06 | 0.04 | 0.02 | 0.02 | 0.01 | 0.02 | 0.03 | 0.05 | 0.05 |
| 1 / 7 . | 0.02 | 0.02 | 0.03 | 0.04 | -0.01 | 0.03 | 0.04 | 0.06 | 0.01 | 0.06 | 0.04 | 0.02 | 0.01 | 0.01 | 0.02 | 0.03 | 0.05 | 0.05 |
| 1 / 8 . | 0.01 | 0.01 | 0.03 | 0.04 | 0.00 | 0.03 | 0.04 | 0.06 | 0.01 | 0.06 | 0.04 | 0.02 | 0.02 | 0.01 | 0.03 | 0.04 | 0.05 | 0.05 |
| 1 / 9 . | 0.01 | 0.02 | 0.03 | 0.04 | -0.02 | 0.03 | 0.04 | 0.06 | 0.01 | 0.06 | 0.04 | 0.02 | 0.02 | 0.01 | 0.02 | 0.03 | 0.05 | 0.05 |
| 1 / 10 . | 0.02 | 0.02 | 0.03 | 0.04 | -0.01 | 0.03 | 0.04 | 0.06 | 0.01 | 0.05 | 0.03 | 0.02 | 0.02 | 0.01 | 0.02 | 0.03 | 0.05 | 0.05 |
| 1 / 11 . | 0.01 | 0.02 | 0.03 | 0.04 | 0.01 | 0.03 | 0.04 | 0.06 | 0.01 | 0.06 | 0.04 | 0.02 | 0.02 | 0.01 | 0.02 | 0.03 | 0.05 | 0.05 |
| 1 / 12 . | 0.02 | 0.02 | 0.03 | 0.04 | 0.00 | 0.03 | 0.04 | 0.06 | 0.01 | 0.06 | 0.04 | 0.02 | 0.02 | 0.01 | 0.02 | 0.03 | 0.05 | 0.05 |
| 1 / 13 . | 0.02 | 0.02 | 0.03 | 0.04 | 0.01 | 0.03 | 0.04 | 0.06 | 0.01 | 0.06 | 0.04 | 0.02 | 0.02 | 0.01 | 0.02 | 0.03 | 0.05 | 0.05 |
| 1 / 14 . | 0.02 | 0.02 | 0.03 | 0.04 | -0.02 | 0.03 | 0.04 | 0.06 | 0.01 | 0.06 | 0.04 | 0.02 | 0.02 | 0.01 | 0.03 | 0.03 | 0.05 | 0.05 |
| 1 / 15 . | 0.01 | 0.02 | 0.03 | 0.04 | 0.01 | 0.03 | 0.04 | 0.06 | 0.01 | 0.06 | 0.04 | 0.02 | 0.02 | 0.01 | 0.02 | 0.03 | 0.05 | 0.05 |
| 1 / 16 . | 0.01 | 0.01 | 0.03 | 0.04 | -0.01 | 0.03 | 0.04 | 0.06 | 0.01 | 0.05 | 0.04 | 0.02 | 0.02 | 0.01 | 0.03 | 0.03 | 0.05 | 0.05 |
| 1 / 17 . | 0.02 | 0.02 | 0.03 | 0.04 | 0.00 | 0.03 | 0.04 | 0.06 | 0.01 | 0.06 | 0.04 | 0.02 | 0.02 | 0.01 | 0.02 | 0.03 | 0.05 | 0.05 |
| 1 / 18 . | 0.02 | 0.02 | 0.03 | 0.04 | 0.01 | 0.03 | 0.04 | 0.06 | 0.01 | 0.06 | 0.04 | 0.02 | 0.02 | 0.01 | 0.02 | 0.03 | 0.05 | 0.06 |
| 1 / 19 . | 0.01 | 0.02 | 0.03 | 0.04 | 0.00 | 0.03 | 0.04 | 0.06 | 0.01 | 0.05 | 0.04 | 0.02 | 0.02 | 0.01 | 0.02 | 0.00 | 0.05 | 0.05 |
| 1 / 20 . | 0.01 | 0.02 | 0.03 | 0.04 | 0.01 | 0.02 | 0.04 | 0.05 | 0.01 | 0.05 | 0.04 | 0.02 | 0.02 | 0.01 | 0.02 | 0.03 | 0.05 | 0.06 |
| 1 / 21 . | 0.02 | 0.02 | 0.03 | 0.04 | 0.00 | 0.03 | 0.04 | 0.06 | 0.01 | 0.05 | 0.04 | 0.02 | 0.02 | 0.01 | 0.02 | 0.03 | 0.05 | 0.05 |
| 1 / 22 . | 0.02 | 0.02 | 0.04 | 0.04 | 0.01 | 0.03 | 0.04 | 0.06 | 0.01 | 0.05 | 0.04 | 0.02 | 0.02 | 0.01 | 0.02 | 0.03 | 0.05 | 0.05 |
| 1 / 23 . | 0.02 | 0.02 | 0.03 | 0.04 | 0.00 | 0.03 | 0.04 | 0.06 | 0.01 | 0.06 | 0.04 | 0.02 | 0.02 | 0.01 | 0.03 | 0.04 | 0.05 | 0.05 |
| 1 / 24 . | 0.02 | 0.02 | 0.04 | 0.04 | 0.00 | 0.03 | 0.04 | 0.06 | 0.01 | 0.05 | 0.04 | 0.02 | 0.02 | 0.01 | 0.02 | 0.03 | 0.05 | 0.06 |
| 1 / 25 . | 0.01 | 0.02 | 0.03 | 0.04 | 0.00 | 0.03 | 0.04 | 0.06 | 0.01 | 0.05 | 0.04 | 0.02 | 0.02 | 0.01 | 0.02 | 0.03 | 0.05 | 0.05 |
| 1 / 26 . | 0.01 | 0.02 | 0.03 | 0.04 | 0.00 | 0.03 | 0.04 | 0.06 | 0.01 | 0.05 | 0.04 | 0.02 | 0.02 | 0.01 | 0.02 | 0.03 | 0.05 | 0.05 |
| 1 / 27 . | 0.01 | 0.01 | 0.03 | 0.04 | 0.01 | 0.03 | 0.04 | 0.06 | 0.01 | 0.06 | 0.04 | 0.02 | 0.02 | 0.01 | 0.02 | 0.04 | 0.05 | 0.06 |
| 1 / 28 . | 0.01 | 0.02 | 0.03 | 0.04 | 0.01 | 0.03 | 0.04 | 0.05 | 0.01 | 0.05 | 0.04 | 0.02 | 0.01 | 0.01 | 0.02 | 0.04 | 0.05 | 0.05 |
| 1 / 29 . | 0.02 | 0.02 | 0.03 | 0.04 | -0.14 | 0.03 | 0.04 | 0.06 | 0.01 | 0.05 | 0.04 | 0.02 | 0.02 | 0.01 | 0.02 | 0.03 | 0.05 | 0.05 |
| 1 / 30 . | 0.02 | 0.02 | 0.03 | 0.04 | -0.01 | 0.03 | 0.04 | 0.06 | 0.01 | 0.05 | 0.04 | 0.02 | 0.02 | 0.01 | 0.02 | 0.03 | 0.05 | 0.05 |
| 1 / 31 . | 0.01 | 0.02 | 0.03 | 0.04 | 0.00 | 0.03 | 0.04 | 0.06 | 0.01 | 0.05 | 0.04 | 0.02 | 0.02 | 0.01 | 0.02 | 0.03 | 0.05 | 0.05 |
| 1 / 32 . | 0.02 | 0.02 | 0.03 | 0.04 | 0.00 | 0.03 | 0.04 | 0.06 | 0.01 | 0.05 | 0.04 | 0.02 | 0.02 | 0.01 | 0.02 | 0.03 | 0.05 | 0.05 |
| 1 / 33 . | 0.01 | 0.02 | 0.03 | 0.04 | 0.00 | 0.03 | 0.04 | 0.06 | 0.01 | 0.06 | 0.04 | 0.02 | 0.02 | 0.01 | 0.02 | 0.03 | 0.05 | 0.05 |
| 1 / 34 . | 0.01 | 0.02 | 0.03 | 0.04 | 0.00 | 0.03 | 0.04 | 0.05 | 0.01 | 0.06 | 0.04 | 0.02 | 0.02 | 0.01 | 0.03 | 0.03 | 0.05 | 0.05 |
| 1 / 35 . | 0.02 | 0.02 | 0.03 | 0.04 | 0.00 | 0.03 | 0.04 | 0.06 | 0.01 | 0.06 | 0.04 | 0.02 | 0.02 | 0.01 | 0.02 | 0.03 | 0.05 | 0.05 |
| 1 / 36 . | 0.01 | 0.02 | 0.03 | 0.04 | 0.00 | 0.03 | 0.04 | 0.06 | 0.01 | 0.06 | 0.04 | 0.02 | 0.02 | 0.01 | 0.03 | 0.03 | 0.05 | 0.05 |
| 1 / 37 . | 0.01 | 0.02 | 0.03 | 0.04 | 0.00 | 0.03 | 0.04 | 0.06 | 0.01 | 0.06 | 0.04 | 0.02 | 0.02 | 0.01 | 0.02 | 0.03 | 0.05 | 0.05 |
| 1 / 38 . | 0.02 | 0.02 | 0.03 | 0.04 | -0.02 | 0.03 | 0.04 | 0.06 | 0.01 | 0.06 | 0.04 | 0.02 | 0.02 | 0.01 | 0.02 | 0.03 | 0.05 | 0.05 |
| 1 / 39 . | 0.01 | 0.02 | 0.03 | 0.04 | -0.02 | 0.03 | 0.04 | 0.06 | 0.01 | 0.05 | 0.04 | 0.02 | 0.02 | 0.01 | 0.02 | 0.03 | 0.05 | 0.05 |
| 1 / 40 . | 0.02 | 0.02 | 0.03 | 0.04 | 0.00 | 0.03 | 0.04 | 0.06 | 0.01 | 0.06 | 0.04 | 0.02 | 0.02 | 0.01 | 0.02 | 0.03 | 0.05 | 0.06 |

**References**

Little, C. T. S., Maslennikov, V. V, Morris, N. J. and Gubanov, A. P. (1999) Two Palaeozoic hydrothermal vent communities from the southern Ural mountains, Russia. *Palaeontology* **42**, 1043–1078.

Maginn, E., Little, C. T. S., Herrington, R. and Mills, R. (2002) Sulphide mineralisation in the deep sea hydrothermal vent polychaete, *Alvinella pompejana*: implications for fossil preservation. *Marine Geology* **181**, 337–356.
